# Prediction of Coronary Artery Disease and Major Adverse Cardiovascular Events Using Clinical and Genetic Risk Scores for Cardiovascular Risk Factors

**DOI:** 10.1053/j.ajkd.2022.01.424

**Published:** 2022-07-13

**Authors:** Julia Ramírez, Stefan van Duijvenboden, William J. Young, Andrew Tinker, Pier D. Lambiase, Michele Orini, Patricia B. Munroe

**Affiliations:** Clinical Pharmacology and Precision Medicine Deparment, William Harvey Research Institute, Barts and The London School of Medicine and Dentistry, Queen Mary University of London, London, United Kingdom (J.R., S.v.D., W.J.Y., A.T., P.B.M.).; Electronic Engineering and Communications Department, Aragon Institute of Engineering Research, University of Zaragoza, Spain and CIBER's Bioengineering, Biomaterials and Nanomedicine, Spain. (J.R.).; Institute of Cardiovascular Science, University College London, London, United Kingdom (S.v.D., P.D.L., M.O.).; Barts Heart Centre, St Bartholomew’s Hospital, London, United Kingdom (W.J.Y., P.D.L., M.O.).; NIHR Barts Cardiovascular Biomedical Research Centre, Barts and The London School of Medicine and Dentistry, Queen Mary University of London, United Kingdom (A.T., P.B.M.).

**Keywords:** coronary artery disease, epidemiology, genetic predisposition, genetic screening, risk factors

## Abstract

Coronary artery disease (CAD) and major adverse cardiovascular events (MACE) are the leading causes of death in the general population, but risk stratification remains suboptimal. CAD genetic risk scores (GRSs) predict risk independently from clinical tools, like QRISK3. We assessed the added value of GRSs for a variety of cardiovascular traits (CV GRSs) for predicting CAD and MACE and tested their early-life screening potential by comparing against the CAD GRS only.

**Methods:**

We used data from 379 581 participants in the UK Biobank without known cardiovascular conditions (follow-up, 11.3 years; 3.3% CAD cases and 5.2% MACE cases). In a training subset (50%) we built 3 scores: QRISK3; QRISK3 and an established CAD GRS; and QRISK3, the CAD GRS and the CV GRSs. In an independent subset (50%), we evaluated each score’s performance using the concordance index, odds ratio and net reclassification index. We then repeated the analyses without considering QRISK3.

**Results:**

For CAD, the combination of QRISK3 and the CAD GRS had a better performance than QRISK3 alone (concordance index, 0.766 versus 0.753; odds ratio, 5.47 versus 4.82; net reclassification index, 7.7%). Adding the CV GRSs did not significantly improve risk stratification. When only looking at genetic information, the combination of CV GRSs and the CAD GRS had a better performance than the CAD GRS alone (concordance index, 0.637 versus 0.625; odds ratio, 2.17 versus 2.07; net reclassification index, 3.3%). Similar results were obtained for MACE.

**Conclusions:**

In individuals without known cardiovascular disease, the inclusion of CV GRSs to a clinical tool and an established CAD GRS does not improve CAD or MACE risk stratification. However, their combination only with the CAD GRS increases prediction performance indicating potential use in early-life screening before the advanced development of conventional cardiovascular risk factors.

Cardiovascular mortality is the main cause of death in the general population,^1^ with a global estimated cost expected to be $1044 billion by 2030.^2^ Coronary artery disease (CAD) and, more generally, major adverse cardiovascular events (MACE) are the leading causes of cardiovascular morbidity and mortality worldwide.^3,4^ Therefore, early identification of individuals at high risk is essential for primary prevention.

Validated clinical risk scores, like QRISK3,^5^ Framingham,^6^ or ASSIGN,^7^ assess long-term cardiovascular risk by combining information from traditional risk factors and, therefore, can be utilized to identify subgroups at risk. More recently, genome-wide association studies have discovered important genetic associations with CAD.^8^ Genetic risk scores (GRSs) combining these genetic associations reflect an individual’s genetic predisposition for CAD and have reported a strong association with CAD and MACE risk. However, their improvement with respect to conventional risk factors or clinical scores is still unclear, with some studies showing an enhanced risk stratification^9–11^ and others only reporting a benefit early in life when information on the risk factors is still unknown.^12–14^

Given that most CAD and MACE risk factors are heritable, with previous publications reporting significantly associated genetic variants, and a shared genetic architecture with cardiovascular risk,^10,15–33^ we hypothesized that the inclusion of GRSs for cardiovascular risk factors may further improve CAD and MACE risk stratification.

In this study, we performed a thorough and detailed assessment of the CAD and MACE risk stratification value of multiple GRSs for cardiovascular risk factors in a middle-aged population without known cardiovascular disease. First, we assessed their performance when integrated with QRISK3 and a CAD GRS.^10^ We then tested their potential for early-life screening by comparing them with the CAD GRS only.^10^

## Methods

**Figure 1.**
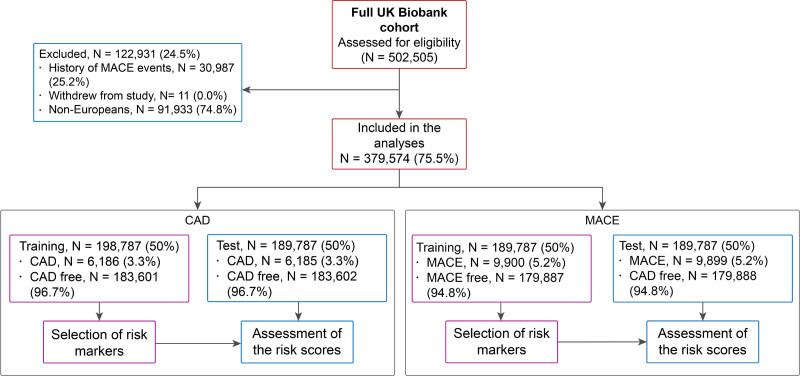
Flowchart indicating the number of individuals included in the study and the partition into training and test for coronary artery disease (CAD) and major adverse cardiovascular event (MACE) end points.

The experimental design of the study is shown in Figure 1. The UK Biobank is a prospective study of 502 505 individuals, comprising relatively even numbers of men and women aged 40 to 69 years at recruitment (2006–2008). Individuals were excluded if they were admitted to the hospital due to any of the *International Classification of Diseases, Tenth Revision*, codes in Table S1 prior recruitment. The primary end point of this study was CAD-related events, defined as CAD mortality or admission to hospital with a CAD diagnosis (*International Classification of Diseases, Tenth Revision*, codes I21–I23; Table S2). The secondary end point was MACE events. Methods describing the study population, risk factors included in the analyses, derivation of risk models, and evaluation of risk scores are available in the Supplemental Material. The UK Biobank study has approval from the North West Multi-Centre Research Ethics Committee, and all participants provided informed consent.^34^ Data used in this study were part of the UK Biobank application number 8256, and anonymized data and materials generated in this work have been returned to the UK Biobank and can be accessed per request.

## Results

### Characteristics of the Study Population

During follow-up, there were 6186 CAD events (3.3%) and 9900 MACE events (5.2%) in each respective training set (similar prevalence in the corresponding test sets; Figure 1). Differences in QRISK3 and the GRSs between the CAD and CAD-free and between the MACE and MACE-free groups are shown in Table S3. A detailed list of traits for which we derived a GRS is described in Table S4.

### Performance of a Score Combining QRISK3, CAD GRS, and GRSs for Cardiovascular Risk Factors

In univariable logistic regression analyses, QRISK3, as well as the GRSs for CAD, body mass index (BMI), C-reactive protein, systolic blood pressure, diastolic blood pressure (DBP), pulse pressure (PP), type 2 diabetes, LDL (low-density lipoprotein) cholesterol, HDL (high-density lipoprotein) cholesterol, triglycerides, resting T-peak-to-T-end interval (Tpe), atrial fibrillation, and heart failure (HF) were significantly associated with CAD (Table 1). As described in Supplemental Methods, score 1 was QRISK3. Score 2 comprised QRISK3, the CAD GRS,^10^ the genetic array, and the fifth and ninth principal components, as they independently contributed to CAD risk (Table 2). Score 3 additionally included the GRSs for BMI, DBP, type 2 diabetes, HF, LDL cholesterol, PP, and resting Tpe—the GRSs that remained significantly associated with CAD (Table 2).

**Table 1.**
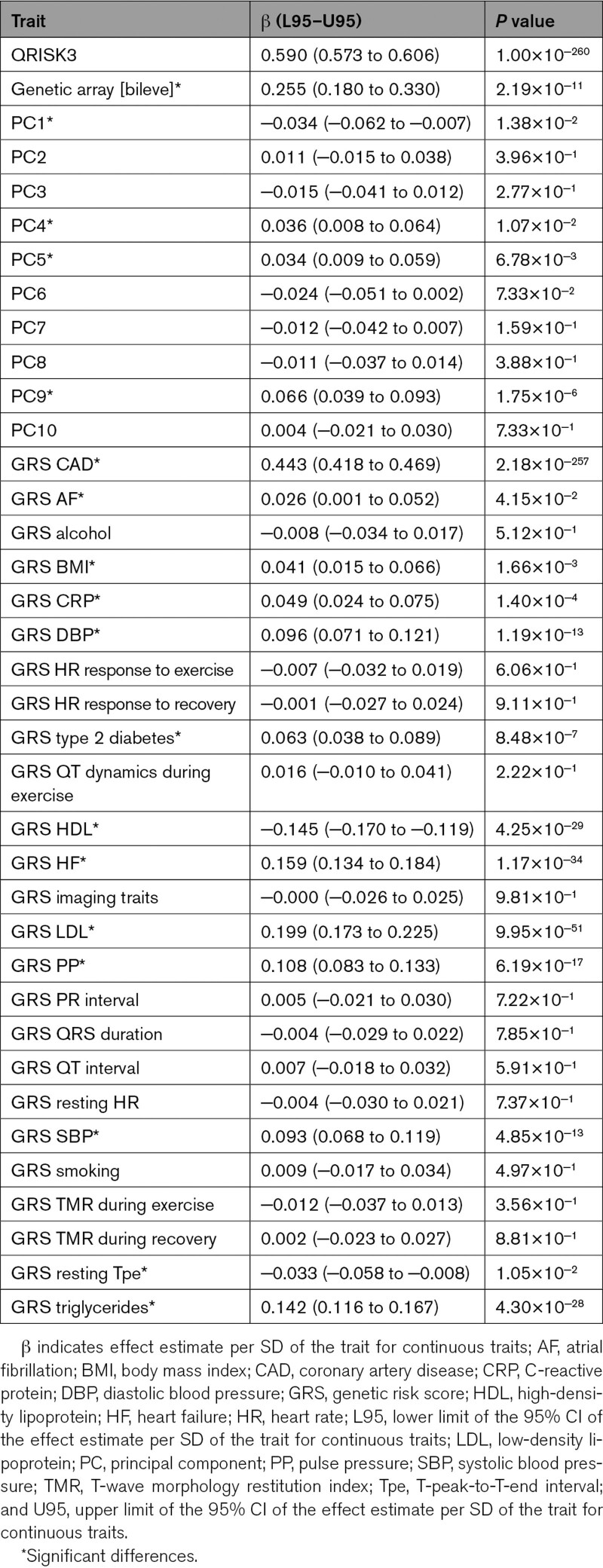
Univariable Logistic Regression Analyses for CAD

**Table 2.**
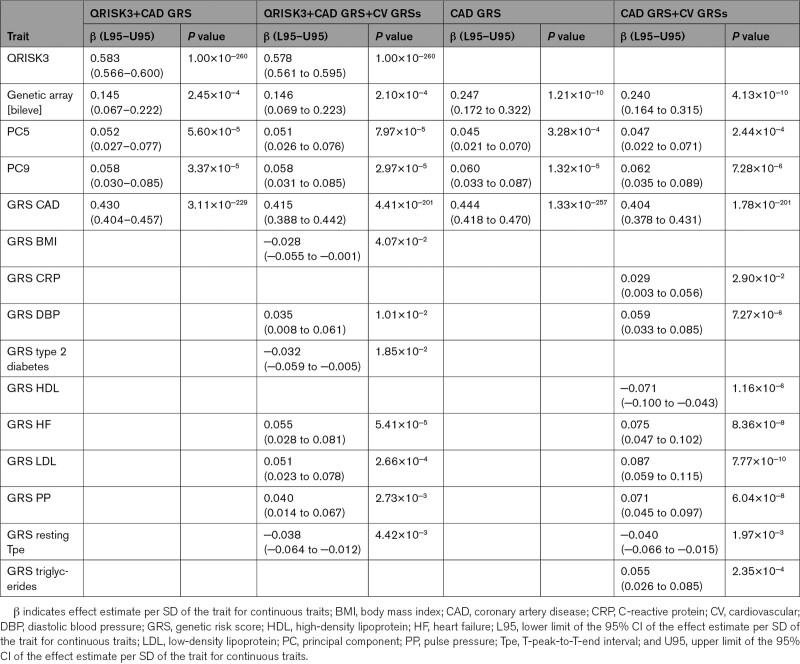
Risk Factors in the Scores for CAD

**Figure 2.**
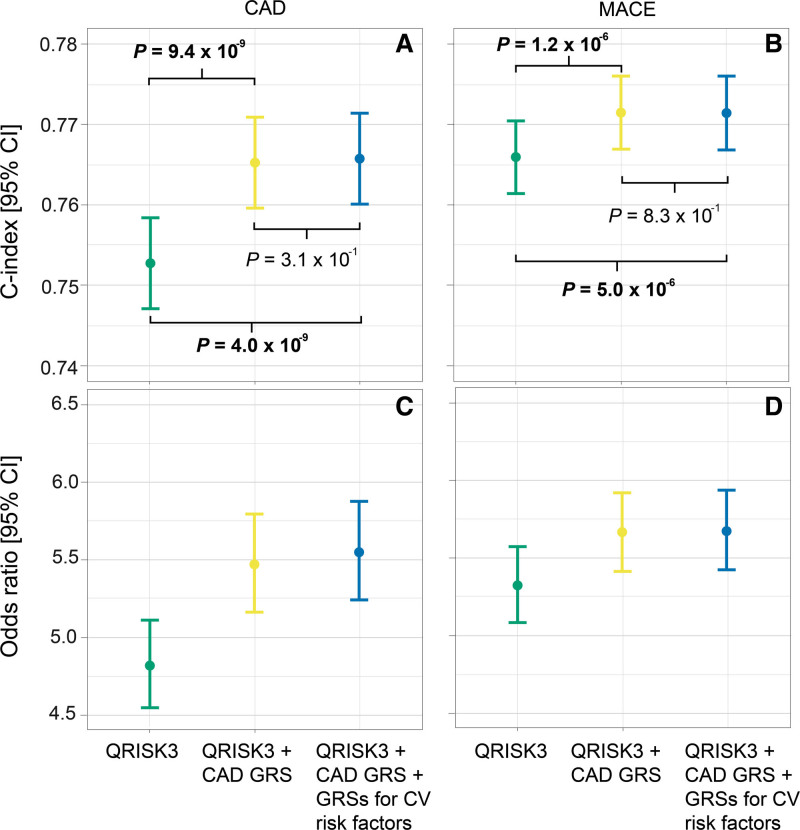
**Performance of genetic risk scores (GRSs) for cardiovascular (CV) risk factors when combined with QRISK3 and a validated coronary artery disease (CAD) GRS.** Concordance indices (C indices) are shown in **A** and **B** for CAD and major adverse cardiovascular events (MACE), respectively. **C** and **D** show the odds ratio of individuals in the high- vs low-risk groups for CAD and MACE, respectively. Yellow (QRISK3+CAD GRS) and blue (QRISK3+CAD GRS+GRSs for CV risk factors) scores are also adjusted for the genetic array and the first 10 principal components.

Figure 2A shows the concordance index (C index) of the 3 scores in the test set when classifying CAD risk. The C index for QRISK3 was 0.753 (95% CI, 0.747–0.758). The C index progressively increased after adding the CAD GRS (C index, 0.765 [0.760–0.771]), being significantly higher than the C index for QRISK3 (*P*=9.4×10^−9^). However, the addition of the GRSs for multiple cardiovascular risk factors did not further increase the C index (0.766 [0.760–0.772]), showing a nonsignificant difference with respect to score 2 (*P*=3.1×10^−1^). Concordantly, the odds ratio (OR) and 95% CI for individuals in the high-risk group versus those in the low-risk group progressively increased from 4.82 (4.55–5.11) for QRISK3 to 5.47 (5.16–5.80) for QRISK3+CAD GRS (Figure 2C). However, there was no further improvement after adding the GRSs for cardiovascular risk factors (OR, 5.55 [CI, 5.24–5.88]). The overall mean net reclassification index (NRI) was 7.7% for score 2 versus score 1 (Table S5).

For MACE, score 2 included QRISK3 (score 1), the CAD GRS, the genetic array, and the ninth principal component (Tables 3 and 4). Score 3 additionally included the GRSs for atrial fibrillation, BMI, DBP, heart rate response to exercise, type 2 diabetes, HDL cholesterol, HF, imaging traits, PP, and resting Tpe (Table 4). Inclusion of the CAD GRS improved the risk stratification provided by QRISK3 alone, but the addition of the GRSs for multiple cardiovascular risk factors did not show a significant benefit (Figure 2B and 2D). The overall mean NRI value for score 2 versus score 1 (QRISK3) was 3.9% (Table S6).

**Table 3.**
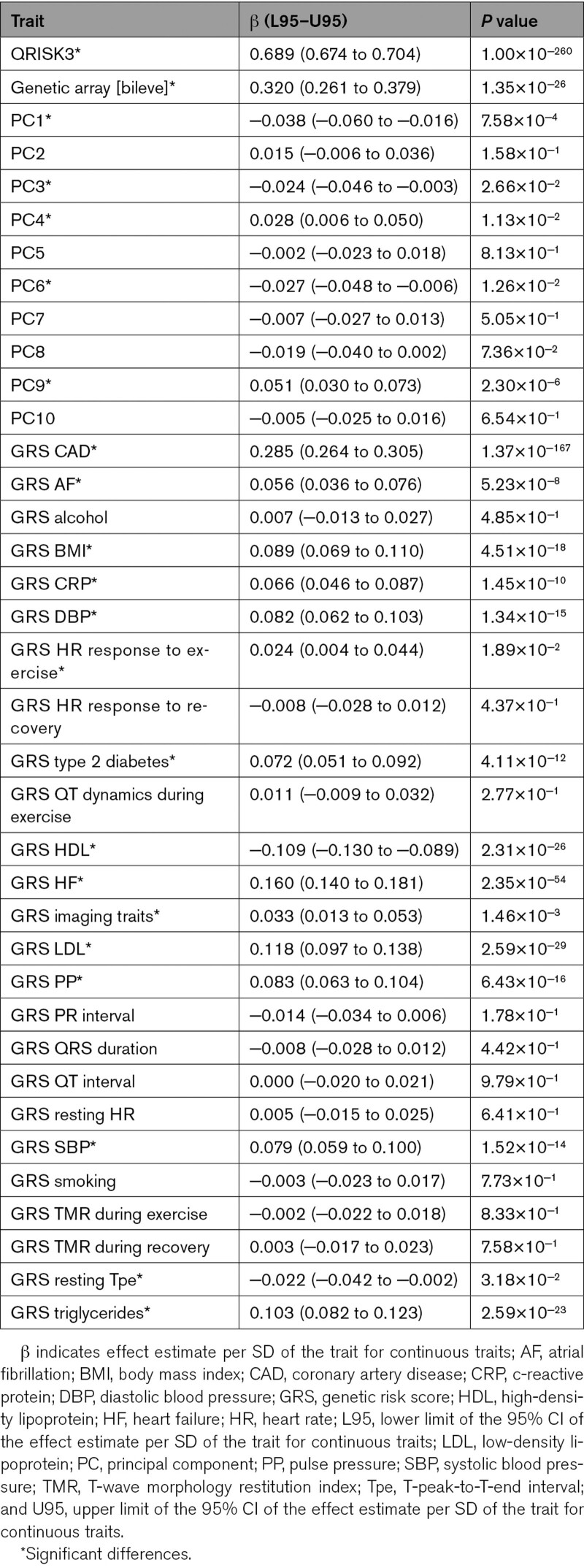
Univariable Logistic Regression Analyses for Major Adverse Cardiovascular Events

**Table 4.**
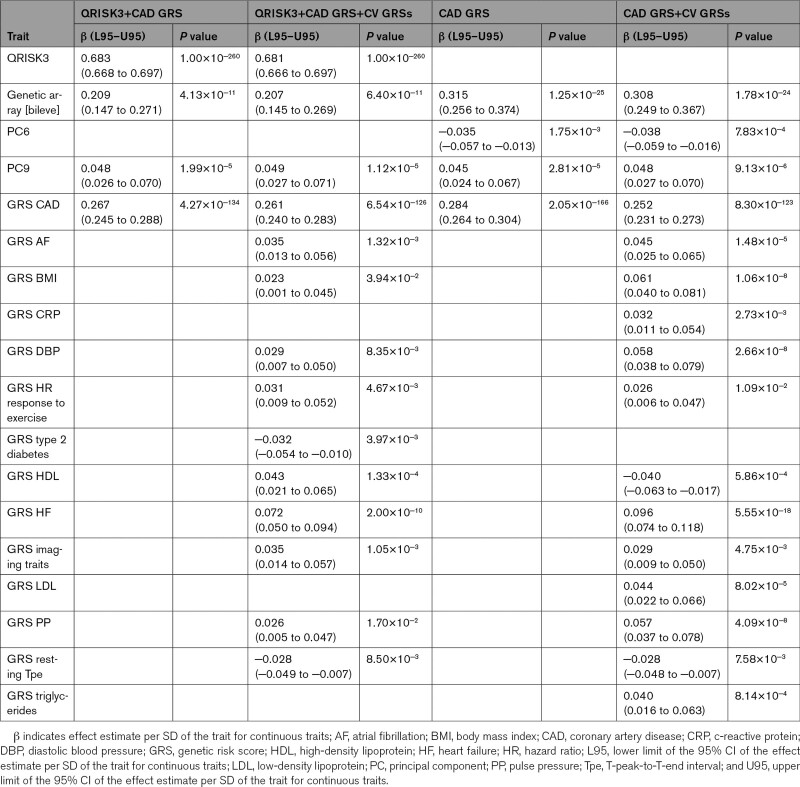
Risk Factors in the Scores for MACE

### Performance of a Score Combining Only CAD GRS and GRSs for Cardiovascular Risk Factors

**Figure 3.**
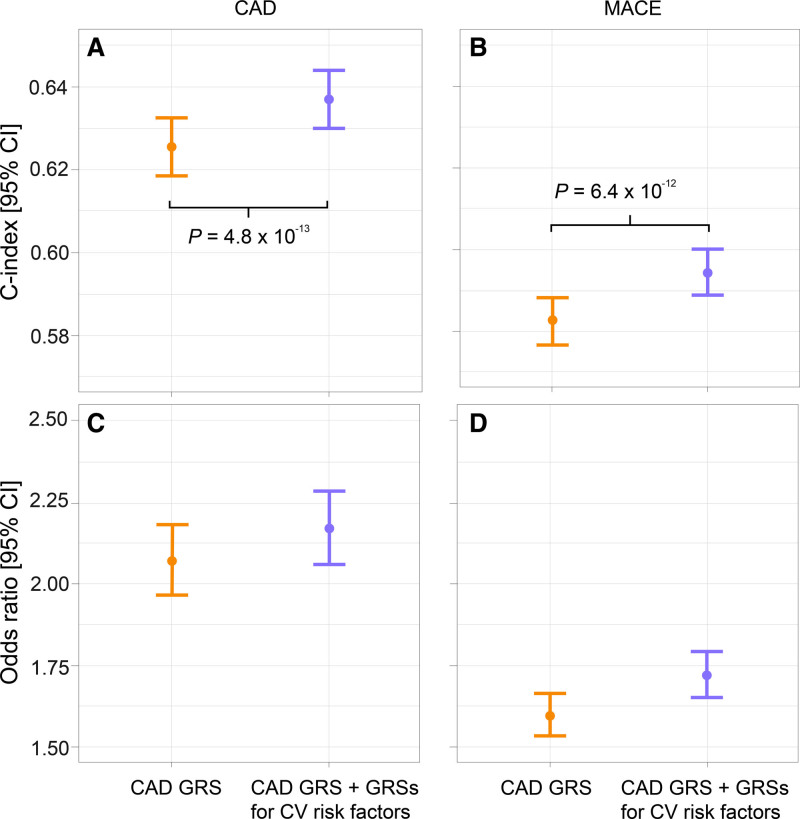
**Performance of genetic risk scores (GRSs) for cardiovascular (CV) risk factors when combined with a validated coronary artery disease (CAD) GRS.** Concordance indices (C indices) are shown in **A** and **B** for CAD and major adverse cardiovascular events (MACE), respectively. **C** and **D** show the odds ratio of individuals in the high- vs low-risk groups for CAD and MACE, respectively. Both scores are also adjusted for the genetic array and the first 10 principal components.

When QRISK3 was not taken into account, score 4 included the CAD GRS, the genetic array, and the fifth and ninth principal components (Table 2). Score 5 additionally included the GRSs for C-reactive protein, DBP, HDL cholesterol, HF, LDL cholesterol, PP, resting Tpe, and triglycerides (Table 2). Risk stratification improved when combining the CAD GRS with GRSs for multiple cardiovascular risk factors compared with the CAD GRS alone (C index, 0.637 [95% CI, 0.630–0.644] versus 0.625 [95% CI, 0.618–0.633]; *P*=4.8×10^−13^; OR, 2.17 [95% CI, 2.06–2.28] versus 2.07 [95% CI, 1.96–2.18]; Figure 3A and 3C). The overall mean NRI was 3.3% (Table S7).

For MACE, score 4 included the CAD GRS, the genetic array, and the sixth and the ninth principal components (Table 4). Score 5 additionally included the GRSs for atrial fibrillation, BMI, C-reactive protein, DBP, PP, heart rate response to exercise, LDL cholesterol, HDL cholesterol, triglycerides, HF, imaging traits, and resting Tpe (Table 4). Inclusion of the GRSs for multiple cardiovascular risk factors improved the risk stratification provided by the CAD GRS alone (Figure 3B and 3D). The overall mean NRI value was 3.9% (Table S8).

## Discussion

In this study, we evaluated the CAD and MACE risk stratification value of GRSs for multiple cardiovascular risk factors in a middle-aged population of >370 000 individuals without known cardiovascular disease. We first demonstrate that they do not improve the risk stratification provided by a validated clinical score, QRISK3, and a well-calibrated CAD GRS.^10^ We then show their potential added value when using only genetic information.

The combination of QRISK3 and the CAD GRS^10^ showed a significant increment in the CAD risk stratification provided by QRISK3 alone in our study population (with a lower gain for MACE risk stratification), confirming results from previous studies comparing against conventional risk factors^9,10,12,13^ and clinical scores.^9–11,14^ In particular, we observed an OR for individuals in the high-risk group versus those in the low-risk group being ≈13% higher for CAD and a mean NRI value of 7.7% compared with using QRISK3 only (Figure 2C; Table S5). However, the inclusion of GRSs including millions of variants for some cardiovascular risk factors did not further improve CAD or MACE risk stratification. These results expand conclusions from previous studies^12–14^ stating that elevated CAD or MACE risk in middle age is mainly influenced by conventional clinical risk factors, with an additional contribution of CAD genetic susceptibility. Thus, at the moment, inclusion of GRSs for cardiovascular risk factors would not yield a clinically meaningful impact if access to a well-established, comprehensive clinical risk score is available. Future studies leveraging updated GRSs, as genetic data become widely available, as well as information from exome or whole-genome association studies, may change this observation.

When considering genetic information only, we show that the GRSs for cardiovascular risk factors significantly improve CAD and MACE risk stratification (Figure 3). The OR for CAD for individuals in the high-risk group versus those in the low-risk group was ≈5% higher compared with using the CAD GRS only (Figure 3C), with a mean NRI value of 3.3% (Table S7). Using the CAD GRS alone, there would be 15 280 individuals classified as intermediate risk (5%–10%) of a CAD event at the end of follow-up (Table S7) and hence not referred for specific preventive measures. The addition of the GRSs for cardiovascular risk factors would reclassify 405 individuals as high risk (ie, ≥10%) and hence eligible for referral, from which 47 would have a CAD event by the end of the follow-up period in our cohort. Our findings open potential opportunities for testing in young populations before the onset of related comorbidities, enabling earlier primary prevention and lifestyle modifications.^12–14^ Importantly, since GRSs can be measured from birth, they could improve primary prevention strategies by identifying those at the highest risk early, before the onset of clinically measurable risk factors. This would facilitate lifestyle modification and patient education, which has been demonstrated to reduce CAD and MACE events.^35^

Our findings also shed some light into the mechanistic interpretation of CAD and MACE risk. The GRSs for BMI, blood pressure, type 2 diabetes, HF, and resting Tpe independently contributed to CAD risk (Table 2), suggesting that they provide additional information relative to CAD risk that is not entirely captured by QRISK3 or the GRS for CAD. The same GRSs in addition to the GRS for atrial fibrillation and for imaging traits were significantly associated with MACE independently from QRISK3 and the GRS for CAD (Table 4). Although ≈65% of the MACE events overlapped with CAD, this more general grouping allowed us to evaluate the specificity of our findings with CAD risk. Our results suggest that the GRSs for cardiovascular risk factors are contributing to a broad definition of cardiovascular risk, rather than targeting CAD-specific risk pathways in this population. Regarding the GRSs for ECG risk markers, we included them as we hypothesized they would share mechanisms of disease with CAD or MACE, reflecting proarrhythmic electrophysiological mechanisms in the heart (heart rate, conduction, and ventricular repolarization).^22–32^ The GRS for resting Tpe was significantly associated with CAD and MACE risk (Tables 1 and 2), suggesting it shares biological pathways with the risk of developing CAD or MACE.

Our study has strengths and limitations. The main strength is the use of one of the largest cohorts currently available with detailed phenotypic and genetic data in a population with no history of cardiovascular events and long follow-up. In addition, the selection of risk factors into the scores and testing of their risk stratification value was performed in genetically unrelated populations (training and test), thus minimizing the risk of overfitting. However, validation of these findings in other cohorts will provide support for generalizability to other cohorts with different characteristics (ie, ethnicity or underlying condition). The study is limited to the UK Biobank cohort, known to have a healthy volunteer selection bias.^36^ Second, the UK Biobank–derived GRSs are associated with birth location within the UK Biobank, and major health outcomes have been reported to be geographically structured,^37^ potentially yielding biased associations. Third, genetic variants selected for inclusion in many of the GRSs, as well as the effect sizes, were obtained from GWASs that included individuals from the UK Biobank, and this might have entailed a risk of overfitting. Fourth, the NRI results might change based on different risk thresholds for treatment initiation, so our results should be interpreted according to the NRI calculation described here. Fifth, stepwise regression has previously shown some limitations,^38^ so future studies using other variable selection algorithms^39^ would be of value. Finally, we only included individuals of the European ancestry; therefore, similar studies are necessary in cohorts with different ancestries.

In conclusion, in a middle-aged general population, GRSs for multiple cardiovascular risk factors do not improve the CAD and MACE risk stratification value provided by QRISK3 and a CAD GRS. However, they show potential when included with a CAD GRS for early-life screening and earlier initiation of primary prevention therapies. From a clinical point of view, these results shed important insights into the use of GRSs in the general population without known cardiovascular disease.

## Article Information

### Acknowledgments

None.

### Sources of Funding

Dr Lambiase is supported by University College London/University College London Hospital Biomedicine National Institute for Health and Care Research. W.J. Young is supported by an Medical Research Council grant MR/R017468/1. Dr Ramírez acknowledges funding from the European Union’s Horizon 2020 Research and Innovation Programme under the Marie Sklodowska-Curie grant agreement number 786833 and from the European Union-NextGenerationEU. The authors wish to acknowledge support by the Medical Research Council grant MR/N025083/1. Drs Tinker and Munroe acknowledge the National Institute for Health and Care Research Cardiovascular Biomedical Research Centre at Barts and Queen Mary University of London, United Kingdom. Drs Orini and Munroe were joint supervisors.

### Disclosures

None.

### Supplemental Material

Supplemental Methods

Tables S1–S8

References 40–47

## Supplementary Material


