# Prediction of Coronary Artery Disease and Major Adverse Cardiovascular Events using Clinical and Genetic Risk Scores for Cardiovascular Risk Factors

**DOI:** 10.1053/j.ajkd.2022.01.424

**Published:** 2022-03-11

**Authors:** Julia Ramírez, Stefan van Duijvenboden, William J. Young, Andrew Tinker, Pier D. Lambiase, Michele Orini, Patricia B. Munroe

**Affiliations:** 1 Clinical Pharmacology, William Harvey Research Institute, Barts and The London School of Medicine and Dentistry, Queen Mary University of London, London, United Kingdom; 2 Aragon Institute of Engineering Research, University of Zaragoza, Zaragoza, Spain and CIBER’s Bioengineering, Biomaterials and Nanomedicine, Spain; 3 Institute of Cardiovascular Science, University College London, London, United Kingdom; 4 Barts Heart Centre, St Bartholomew’s Hospital, London, United Kingdom; 5 NIHR Barts Cardiovascular Biomedical Research Centre, Barts and The London School of Medicine and Dentistry, Queen Mary University of London, London, United Kingdom

**Keywords:** risk prediction, epidemiology, genetic risk scores, coronary artery disease

## Abstract

**Background:**

Coronary artery disease (CAD) and major adverse cardiovascular events (MACE) are leading causes of death in the general population, but risk stratification remains suboptimal. CAD genetic risk scores (GRSs) predict risk independently from clinical tools, like QRISK3. We assessed the added value of GRSs for a variety of cardiovascular traits (CV GRSs) for predicting CAD and MACE and tested their early-life screening potential by comparing against the CAD GRS only.

**Methods:**

We used data from 379,581 participants in the UK Biobank without known cardiovascular conditions (follow-up 11.3 years, 3.3% CAD cases, 5.2% MACE cases). In a training subset (50%) we built three scores: QRISK3; QRISK3 and an established CAD GRS; and QRISK3, the CAD GRS and the CV GRSs. In an independent subset (50%), we evaluated each score’s performance using the concordance (C) index, odds ratio (ORs) and net reclassification index (NRI). We then repeated the analyses without considering QRISK3.

**Results:**

For CAD, the combination of QRISK3 and the CAD GRS had a better performance than QRISK3 alone (C index of 0.766 versus 0.753, OR of 5.47 versus 4.82, NRI of 7.7%). Adding the CV GRSs did not significantly improve risk stratification. When only looking at genetic information, the combination of CV GRSs and the CAD GRS had a better performance than the CAD GRS alone (C index of 0.637 versus 0.625, OR of 2.17 versus 2.07, NRI of 3.3%). Similar results were obtained for MACE.

**Conclusions:**

In individuals without known cardiovascular disease, the inclusion of CV GRSs to a clinical tool and an established CAD GRS does not improve CAD or MACE risk stratification. However, their combination only with the CAD GRS increases prediction performance indicating potential use in early-life screening before the advanced development of conventional CV risk factors.

## Nonstandard Abbreviations and Acronyms

AF: Atrial fibrillation
BMI: Body mass index
C index: concordance index
CAD: Coronary artery disease
CI: Confidence interval
CRP: C-reactive protein
DBP: Diastolic blood pressure
GRS: Genetic risk score
HDL: High-density lipoprotein cholesterol
HF: Heart failure
OR: Odds ratio
ICD-10: International Classification of Diseases, Tenth Revision
LDL: Low-density lipoprotein cholesterol
MACE: Major adverse cardiovascular events
SBP: Systolic blood pressure
SCD: Sudden cardiac death

## Introduction

Cardiovascular (CV) mortality is the main cause of death in the general population^1^, with a global estimated cost expected to be $1,044 billion by 2030^2^. Coronary artery disease (CAD) and, more generally, major adverse cardiovascular events (MACE) are leading causes of CV morbidity and mortality worldwide ^3, 4^. Therefore, early identification of individuals at high risk is essential for primary prevention.

Validated clinical risk scores, like QRISK3^5^, Framingham^6^ or ASSIGN^7^, assess long-term cardiovascular risk by combining information from traditional risk factors and, therefore, can be utilised to identify subgroups at risk. More recently, genome-wide association studies have discovered important genetic associations with CAD^8^. Genetic risk scores (GRSs) combining these genetic associations reflect an individual’s genetic predisposition for CAD and have reported a strong association with CAD and MACE risk. However, their improvement with respect to conventional risk factors or clinical scores is still unclear, with some studies showing an enhanced risk stratification^9–11^, and others only reporting a benefit early in life when information on the risk factors is still unknown^12–14^.

Given that most CAD and MACE risk factors are heritable, with previous publications reporting significantly associated genetic variants, and a shared genetic architecture with CV risk ^10,15–33^, we hypothesised that the inclusion of GRSs for CV risk factors may further improve CAD and MACE risk stratification.

In this study, we performed a thorough and detailed assessment of the CAD and MACE risk stratification value of multiple GRSs for CV risk factors in a middle-age population without known CV disease. First, we assessed their performance when integrated with QRISK3 and a CAD GRS^10^. We, then, tested their potential for early life screening by comparing them with the CAD GRS only^10^.

## Methods

**Figure 1:**
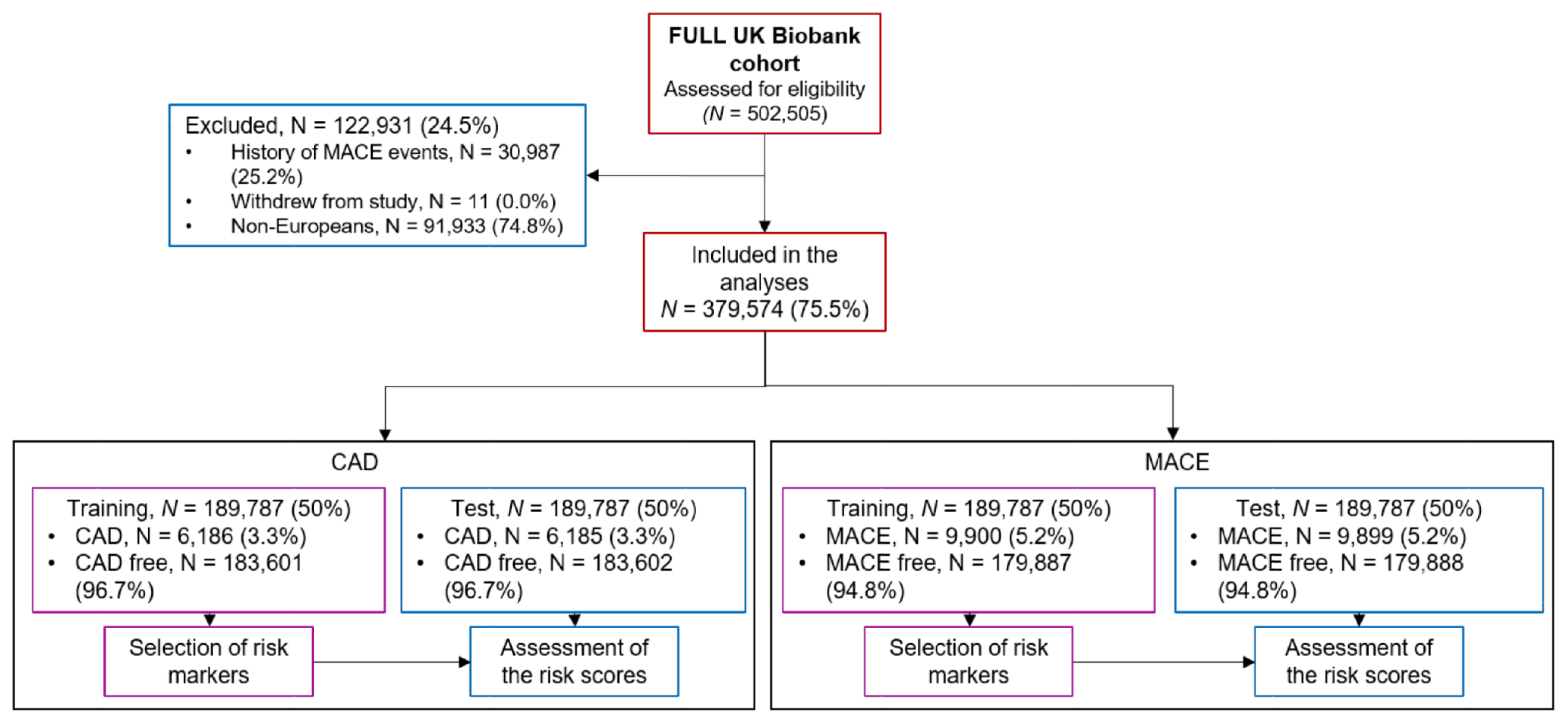
Flow diagram indicating the number of individuals included in the study, and the partition into training and test for CAD and MACE endpoints. CAD, coronary artery disease; MACE, major adverse cardiovascular events

The experimental design of the study is shown in Figure 1. UK Biobank is a prospective study of 502,505 individuals, comprising relatively even numbers of men and women aged 40 to 69 years old at recruitment (2006 - 2008). Individuals were excluded if they were admitted to hospital due to any of the International Classification of Diseases, Tenth Revision (ICD-10) codes in Supplemental Table I prior recruitment. The primary endpoint of this study was CAD related events, defined as CAD mortality or admission to hospital with a CAD diagnosis (ICD10 codes I21-I23, Supplemental Table II). The secondary endpoint was MACE events. Methods describing the study population, risk factors included in the analyses, derivation of risk models, and evaluation of risk scores are available in the Supplemental Material. The UK Biobank study has approval from the North West Multi-Centre Research Ethics Committee, and all participants provided informed consent^34^. Data used in this study were part of UK Biobank application number 8256 and anonymised data and materials generated in this work have been returned to UK Biobank and can be accessed per request.

## Results

### Characteristics of the study population

During follow-up, there were 6,186 CAD events (3.3%) and 9,900 MACE events (5.2%) in each respective training set (similar prevalence in the corresponding test sets, Figure 1). Differences in QRISK3 and the GRSs between the CAD and CAD-free and between the MACE and MACE-free groups are shown in Supplemental Table III. A detailed list of traits for which we derived a GRS is described in Supplemental Table IV.

### Performance of a score combining QRISK3, CAD GRS and GRSs for CV risk factors

In Univariable logistic regression analyses, QRISK3, as well as the GRSs for CAD, body mass index (BMI), c-reactive protein (CRP), systolic blood pressure (SBP), diastolic blood pressure (DBP), pulse pressure (PP), type 2 diabetes, low-density lipoprotein (LDL) cholesterol, high-density lipoprotein (HDL) cholesterol, triglycerides, resting T-peak-to-T-end (Tpe), atrial fibrillation (AF) and heart failure (HF) were significantly associated with CAD (Table 1). As described in Supplemental Methods, score 1 was QRISK3. Score 2 comprised QRISK3, the CAD GRS^10^, the genetic array and the fifth and ninth principal components, as they independently contributed to CAD risk (Table 2). Score 3 additionally included the GRSs for BMI, DBP, type 2 diabetes, HF, LDL cholesterol, PP and resting Tpe, the GRSs that remained significantly associated with CAD (Table 2).

**Figure 2:**
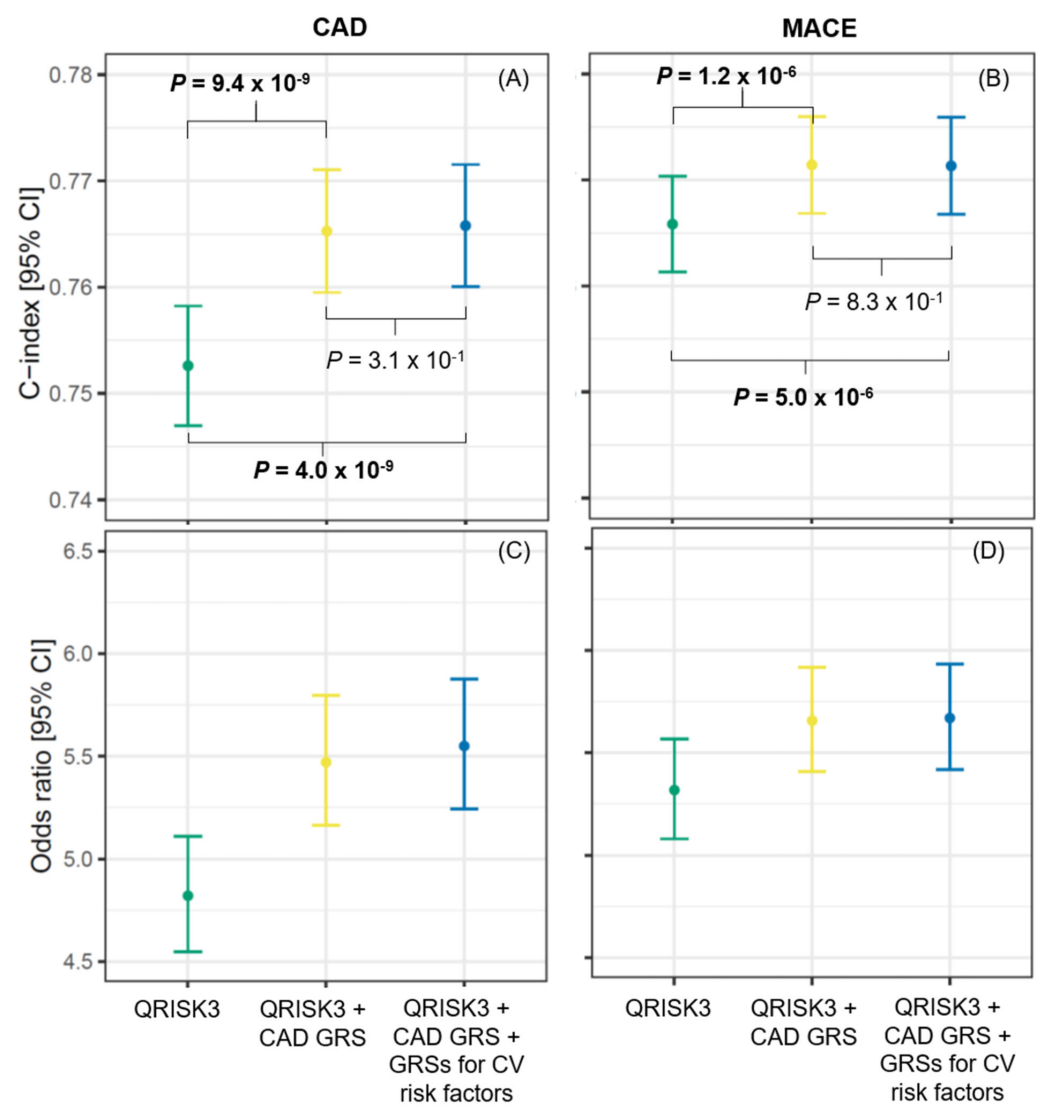
Performance of GRSs for CV risk factors when combined with QRISK3 and a validated CAD GRS. C-indices are shown in panels (A) and (B) for CAD and MACE, respectively. Panels (C) and (D) show the odds ratio of individuals in the high- versus low-risk groups for CAD and MACE, respectively. Yellow (QRISK3 + CAD GRS) and blue (QRISK3 + CAD GRS + GRSs for CV risk factors) scores are also adjusted for the genetic array and first 10 principal components. CAD, coronary artery disease; MACE, major adverse cardiovascular events; GRS, genetic risk score; CV, cardiovascular; CI, confidence interval.

Figure 2 (panel A) shows the concordance (C) index of the three scores in the test set when classifying CAD risk. The C index for QRISK3 was 0.753 (95% confidence interval [CI] of 0.747 – 0.758). The C index progressively increased after adding the CAD GRS (C index of 0.765 [0.760 – 0.771]), being significantly higher than the C index for QRISK3 (*P* = 9.4 x 10^-9^). However, the addition of the GRSs for multiple CV risk factors did not further increase the C index (0.766 [0.760 – 0.772]), showing a non-significant difference with respect to score 2 (*P* = 3.1 × 10^-1^). Concordantly, the odds ratio (OR) and 95% CI for individuals in the high-risk group versus those in the low-risk group progressively increased from 4.82 (4.55 – 5.11) for QRISK3 to 5.47 (5.16 – 5.80) for QRISK3 + CAD GRS (Figure 2, panel C). However, there was no further improvement after adding the GRSs for CV risk factors (OR of 5.55, CI of [5.24 – 5.88]). The overall mean net reclassification index (NRI) was 7.7% for score 2 versus score 1 (Supplemental Table V).

For MACE, score 2 included QRISK3 (score 1), the CAD GRS, the genetic array and the ninth principal component (Tables 3 and 4). Score 3 additionally included the GRSs for AF, BMI, DBP, heart rate (HR) response to exercise, type 2 diabetes, HDL cholesterol, HF, imaging traits, PP and resting Tpe (Table 4). Inclusion of the CAD GRS improved the risk stratification provided by QRISK3 alone, but the addition of the GRSs for multiple CV risk factors did not show a significant benefit (Figure 2, panels B and D). The overall mean NRI value for score 2 versus score 1 (QRISK3) was 3.9% (Supplemental Table VI).

### Performance of a score combining only CAD GRS and GRSs for CV risk factors

**Figure 3:**
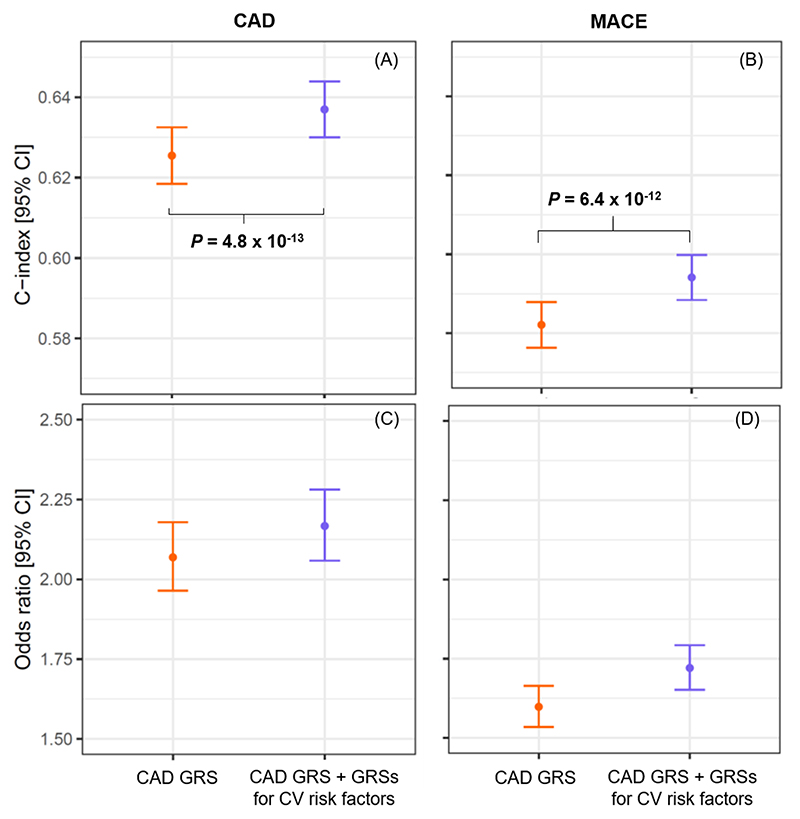
Performance of GRSs for CV risk factors when combined with a validated CAD GRS. C-indices are shown in panels (A) and (B) for CAD and MACE, respectively. Panels (C) and (D) show the odds ratio of individuals in the high- versus low-risk groups for CAD and MACE, respectively. Both scores are also adjusted for the genetic array and first 10 principal components. CAD, coronary artery disease; MACE, major adverse cardiovascular events; GRS, genetic risk score; CV, cardiovascular; CI, confidence interval.

When QRISK3 was not taken into account, score 4 included the CAD GRS, the genetic array, and the fifth and ninth principal components (Table 2). Score 5 additionally included the GRSs for CRP, DBP, HDL cholesterol, HF, LDL cholesterol, PP, resting Tpe and triglycerides (Table 2). Risk stratification improved when combining the CAD GRS with GRSs for multiple CV risk factors compared to the CAD GRS alone (C index of 0.637 [95% CI 0.630 – 0.644] versus 0.625 [95% CI 0.618 – 0.633], *P* = 4.8 × 10^-13^; and OR of 2.17 [95% CI 2.06 – 2.28] versus 2.07 [95% CI 1.96 – 2.18], Figure 3, panels A and C). The overall mean NRI was 3.3% (Supplemental Table VII).

For MACE, score 4 included the CAD GRS, the genetic array, the sixth and the ninth principal components (Table 4). Score 5 additionally included the GRSs for AF, BMI, CRP, DBP, PP, HR response to exercise, LDL cholesterol, HDL cholesterol, triglycerides, HF, imaging traits and resting Tpe (Table 4). Inclusion of the GRSs for multiple CV risk factors improved the risk stratification provided by the CAD GRS alone (Figure 3, panels B and D). The overall mean NRI value was 3.9% (Supplemental Table VIII).

## Discussion

In this study, we evaluated the CAD and MACE risk stratification value of GRSs for multiple CV risk factors in a middle-age population of >370,000 individuals without known cardiovascular disease. We, first, demonstrate that they do not improve the risk stratification provided by a validated clinical score, QRISK3, and a well-calibrated CAD GRS^10^. We, then, show, their potential added value when using only genetic information.

The combination of QRISK3 and the CAD GRS^10^ showed a significant increment in the CAD risk stratification provided by QRISK3 alone in our study population (with a lower gain for MACE risk stratification), confirming results from previous studies comparing against conventional risk factors^9, 10, 12, 13^ and clinical scores^9–11, 14^. In particular, we observed an OR for individuals in the high-risk group versus those in the low-risk group being ~13% higher for CAD and a mean NRI value of 7.7% compared to using QRISK3 only (Figure 2C, Supplemental Table V). However, the inclusion of GRSs including millions of variants for some CV risk factors did not further improve CAD or MACE risk stratification. These results expand conclusions from previous studies^12–14^ stating that elevated CAD or MACE risk in middle age is mainly influenced by conventional clinical risk factors, with an additional contribution of CAD genetic susceptibility. Thus, at the moment, inclusion of GRSs for CV risk factors would not yield a clinically meaningful impact if access to a well-established, comprehensive clinical risk score is available. Future studies leveraging updated GRSs, as genetic data becomes widely available as well as information from exome or whole-genome association studies, may change this observation.

When considering genetic information only, we show that the GRSs for CV risk factors significantly improve CAD and MACE risk stratification (Figure 3). The OR for CAD for individuals in the high-risk group versus those in the low-risk group was ~5% higher compared to using the CAD GRS only (Figure 3C), with a mean NRI value of 3.3% (Supplemental Table VII). Using the CAD GRS alone there would be 15,280 individuals classified as intermediate risk (5% - 10%) of a CAD event at the end of follow-up (Supplemental Table VII), and, hence, not referred for specific preventive measures. The addition of the GRSs for CV risk factors would reclassify 405 individuals as high-risk (i.e. ≥10%), and, hence, eligible for referral, from which 47 would have a CAD event by the end of the follow-up period in our cohort. Our findings open potential opportunities for testing in young populations before the onset of related co-morbidities, enabling earlier primary prevention and lifestyle modifications^12–14^. Importantly, since GRSs can be measured from birth, they could improve primary prevention strategies by identifying those at highest risk early, before the onset of clinically measurable risk factors. This would facilitate lifestyle modification and patient education, which has been demonstrated to reduce CAD and MACE events^35^.

Our findings also shed some light into the mechanistic interpretation of CAD and MACE risk. The GRSs for BMI, blood pressure, type 2 diabetes, HF and resting Tpe independently contributed to CAD risk (Table 2), suggesting that they provide additional information relative to CAD risk that is not entirely captured by QRISK3 or the GRS for CAD. The same GRSs in addition to the GRS for AF and for imaging traits were significantly associated with MACE independently from QRISK3 and the GRS for CAD (Table 4). Although approximately 65% of the MACE events overlapped with CAD, this more general grouping allowed us to evaluate the specificity of our findings with CAD risk. Our results suggest that the GRSs for CV risk factors are contributing to a broad definition of CV risk, rather than targeting CAD-specific risk pathways in this population. Regarding the GRSs for ECG risk markers, we included them as we hypothesised they would share mechanisms of disease with CAD or MACE, reflecting pro-arrhythmic electrophysiological mechanisms in the heart (heart rate, conduction and ventricular repolarization)^22–32^. The GRS for resting Tpe was significantly associated with CAD and MACE risk (Tables 1 and 2), suggesting it shares biological pathways with the risk of developing CAD or MACE.

Our study has strengths and limitations. The main strength is the use of one of the largest cohorts currently available with detailed phenotypic and genetic data in a population with no previous history of cardiovascular events and long follow-up. In addition, the selection of risk factors into the scores and testing of their risk stratification value was performed in genetically unrelated populations (training and test), thus minimising the risk of overfitting. However, validation of these findings in other cohorts will provide support for generalizability to other cohorts with different characteristics (i.e. ethnicity or underlying condition). The study is limited to the UK Biobank cohort, known to have a “healthy volunteer” selection bias^36^. Second, UK Biobank-derived GRSs are associated with birth location within UK Biobank, and major health outcomes have been reported to be geographically structured^37^, potentially yielding biased associations. Third, genetic variants selected for inclusion in many of the GRSs, as well as the effect sizes, were obtained from GWASs that included individuals from UK Biobank, and this might have entailed a risk of overfitting. Fourth, the NRI results might change based on different risk thresholds for treatment initiation, so our results should be interpreted according to the NRI calculation described here. Fifth, stepwise regression has previously shown some limitations^38^, so future studies using other variable selection algorithms^39^ would be of value. Finally, we only included individuals of European ancestry; therefore, similar studies are necessary in cohorts with different ancestries.

In conclusion, in a middle age general population, GRSs for multiple CV risk factors do not improve the CAD and MACE risk stratification value provided by QRISK3 and a CAD GRS. However, they show potential when included with a CAD GRS for early-life screening and earlier initiation of primary prevention therapies. From a clinical point of view, these results shed important insights into the use of GRSs in the general population without known cardiovascular disease.

## Supplementary Material

003441 - Supplemental Material

## Figures and Tables

**Table 1:** Univariable logistic regression analyses for CAD

Trait	Beta (L95 - U95)	P
QRISK3	0,590 (0,573 - 0,606)	1,00E-260
**Genetic array [bileve]**	**0,255 (0,180 - 0,330)**	**2,19E-11**
**PC1**	**-0,034 (-0,062--0,007)**	**1,38E-02**
PC2	0,011 (-0,015 - 0,038)	3,96E-01
PC3	-0,015 (-0,041 - 0,012)	2,77E-01
**PC4**	**0,036 (0,008 - 0,064)**	**1,07E-02**
**PC5**	**0,034 (0,009 - 0,059)**	**6,78E-03**
PC6	-0,024 (-0,051 - 0,002)	7,33E-02
PC7	-0,012 (-0,042 - 0,007)	1,59E-01
PC8	-0,011 (-0,037 - 0,014)	3,88E-01
**PC9**	**0,066 (0,039 - 0,093)**	**1,75E-06**
PC10	0,004 (-0,021 - 0,030)	7,33E-01
**GRS CAD**	**0,443 (0,418 - 0,469)**	**2,18E-257**
**GRS AF**	**0,026 (0,001 - 0,052)**	**4,15E-02**
GRS Alcohol	-0,008 (-0,034 - 0,017)	5,12E-01
**GRS BMI**	**0,041 (0,015 - 0,066)**	**1,66E-03**
**GRS CRP**	**0,049 (0,024 - 0,075)**	**1,40E-04**
**GRS DBP**	**0,096 (0,071 - 0,121)**	**1,19E-13**
GRS HR response to exercise	-0,007 (-0,032 - 0,019)	6,06E-01
GRS HR response to recovery	-0,001 (-0,027 - 0,024)	9,11E-01
**GRS type 2 diabetes**	**0,063 (0,038 - 0,089)**	**8,48E-07**
GRS QT dynamics during exercise	0,016 (-0,010 - 0,041)	2,22E-01
**GRS HDL**	**-0,145 (-0,170 - -0,119)**	**4,25E-29**
**GRS HF**	**0,159 (0,134 - 0,184)**	**1,17E-34**
GRS imaging traits	-0,000 (-0,026 - 0,025)	9,81E-01
**GRS LDL**	**0,199 (0,173 - 0,225)**	**9,95E-51**
**GRS PP**	**0,108 (0,083 - 0,133)**	**6,19E-17**
GRS PR interval	0,005 (-0,021 - 0,030)	7,22E-01
GRS QRS duration	-0,004 (-0,029 - 0,022)	7,85E-01
GRS QT interval	0,007 (-0,018 - 0,032)	5,91E-01
GRS resting HR	-0,004 (-0,030 - 0,021)	7,37E-01
**GRS SBP**	**0,093 (0,068 - 0,119)**	**4,85E-13**
GRS smoking	0,009 (-0,017 - 0,034)	4,97E-01
GRS TMR during exercise	-0,012 (-0,037 - 0,013)	3,56E-01
GRS TMR during recovery	0,002 (-0,023 - 0,027)	8,81E-01
**GRS resting Tpe**	**-0,033 (-0,058 - -0,008)**	**1,05E-02**
**GRS Triglycerides**	**0,142 (0,116 - 0,167)**	**4,30E-28**

* AF, atrial fibrillation; Beta, effect estimate per standard deviation of the trait for continuous traits; BMI, body mass index; CAD, coronary artery disease; CRP, c-reactive protein; DBP, diastolic blood pressure; GRS, genetic risk score; HDL, high density lipoprotein cholesterol; HF, heart failure; L95, lower limit of the 95% confidence interval of the effect estimate per standard deviation of the trait for continuous traits; LDL, low density lipoprotein cholesterol; PC, principal component; PP, pulse pressure; SBP, systolic blood pressure; SCD, sudden cardiac death; TMR, T-wave morphology restitution index; Tpe, T-peak-to-T-end interval; U95, upper limit of the 95% confidence interval of the effect estimate per standard deviation of the trait for continuous traits. Significant differences are indicated in bold.

**Table 2:** Risk factors in the scores for CAD

Trait	QRISK3 + CAD GRS		QRISK3 + CAD GRS + CV GRSs		CAD GRS		CAD GRS + CV GRSs	
	Beta (L95 - U95)	P	Beta (L95 - U95)	P	Beta (L95 - U95)	P	Beta (L95 - U95)	P
QRISK3	0,583 (0,566 - 0,600)	1,00E-260	0,578 (0,561 - 0,595)	1,00E-260				
Genetic array [bileve]	0,145 (0,067 - 0,222)	2,45E-04	0,146 (0,069 - 0,223)	2,10E-04	0,247 (0,172 - 0,322)	1,21E-10	0,240 (0,164 - 0,315)	4,13E-10
PC5	0,052 (0,027 - 0,077)	5,60E-05	0,051 (0,026 - 0,076)	7,97E-05	0,045 (0,021 - 0,070)	3,28E-04	0,047 (0,022 - 0,071)	2,44E-04
PC9	0,058 (0,030 - 0,085)	3,37E-05	0,058 (0,031 - 0,085)	2,97E-05	0,060 (0,033 - 0,087)	1,32E-05	0,062 (0,035 - 0,089)	7,28E-06
GRS CAD	0,430 (0,404 - 0,457)	3,11E-229	0,415 (0,388 - 0,442)	4,41E-201	0,444 (0,418 - 0,470)	1,33E-257	0,404 (0,378 - 0,431)	1,78E-201
GRS BMI			-0,028 (-0,055 - - 0,001)	4,07E-02				
GRS CRP							0,029 (0,003 - 0,056)	2,90E-02
GRS DBP			0,035 (0,008 - 0,061)	1,01E-02			0,059 (0,033 - 0,085)	7,27E-06
GRS type 2 diabetes			-0,032 (-0,059 - - 0,005)	1,85E-02				
GRS HDL							-0,071 (-0,100 - - 0,043)	1,16E-06
GRS HF			0,055 (0,028 - 0,081)	5,41E-05			0,075 (0,047 - 0,102)	8,36E-08
GRS LDL			0,051 (0,023 - 0,078)	2,66E-04			0,087 (0,059 - 0,115)	7,77E-10
GRS PP			0,040 (0,014 - 0,067)	2,73E-03			0,071 (0,045 - 0,097)	6,04E-08
GRS resting Tpe			-0,038 (-0,064 - - 0,012)	4,42E-03			-0,040 (-0,066 - - 0,015)	1,97E-03
GRS Triglycerides							0,055 (0,026 - 0,085)	2,35E-04

* AF, atrial fibrillation; Beta, effect estimate per standard deviation of the trait for continuous traits; BMI, body mass index; CAD, coronary artery disease; CRP, c-reactive protein; DBP, diastolic blood pressure; GRS, genetic risk score; HDL, high density lipoprotein cholesterol; HF, heart failure; L95, lower limit of the 95% confidence interval of the effect estimate per standard deviation of the trait for continuous traits; LDL, low density lipoprotein cholesterol; MACE, major adverse cardiovascular events; PC, principal component; PP, pulse pressure; SBP, systolic blood pressure; SCD, sudden cardiac death; TMR, T-wave morphology restitution index; Tpe, T-peak-to-T-end interval; U95, upper limit of the 95% confidence interval of the effect estimate per standard deviation of the trait for continuous traits.

**Table 3:** Univariable logistic regression analyses for MACE

Trait	Beta (L95 - U95)	P
**QRISK3**	**0,689 (0,674 - 0,704)**	**1,00E-260**
**Genetic array [bileve]**	**0,320 (0,261 - 0,379)**	**1,35E-26**
**PC1**	**-0,038 (-0,060 - -0,016)**	**7,58E-04**
PC2	0,015 (-0,006 - 0,036)	1,58E-01
**PC3**	**-0,024 (-0,046 - -0,003)**	**2,66E-02**
**PC4**	**0,028 (0,006 - 0,050)**	**1,13E-02**
PC5	-0,002 (-0,023 - 0,018)	8,13E-01
**PC6**	**-0,027 (-0,048 - -0,006)**	**1,26E-02**
PC7	-0,007 (-0,027 - 0,013)	5,05E-01
PC8	-0,019 (-0,040 - 0,002)	7,36E-02
**PC9**	**0,051 (0,030 - 0,073)**	**2,30E-06**
PC10	-0,005 (-0,025 - 0,016)	6,54E-01
**GRS CAD**	**0,285 (0,264 - 0,305)**	**1,37E-167**
**GRS AF**	**0,056 (0,036 - 0,076)**	**5,23E-08**
GRS Alcohol	0,007 (-0,013 - 0,027)	4,85E-01
**GRS BMI**	**0,089 (0,069 - 0,110)**	**4,51E-18**
**GRS CRP**	**0,066 (0,046 - 0,087)**	**1,45E-10**
**GRS DBP**	**0,082 (0,062 - 0,103)**	**1,34E-15**
**GRS HR response to exercise**	**0,024 (0,004 - 0,044)**	**1,89E-02**
GRS HR response to recovery	-0,008 (-0,028 - 0,012)	4,37E-01
**GRS type 2 diabetes**	**0,072 (0,051 - 0,092)**	**4,11E-12**
GRS QT dynamics during exercise	0,011 (-0,009 - 0,032)	2,77E-01
**GRS HDL**	**-0,109 (-0,130 - -0,089)**	**2,31E-26**
**GRS HF**	**0,160 (0,140 - 0,181)**	**2,35E-54**
**GRS imaging traits**	**0,033 (0,013 - 0,053)**	**1,46E-03**
**GRS LDL**	**0,118 (0,097 - 0,138)**	**2,59E-29**
**GRS PP**	**0,083 (0,063 - 0,104)**	**6,43E-16**
GRS PR interval	-0,014 (-0,034 - 0,006)	1,78E-01
GRS QRS duration	-0,008 (-0,028 - 0,012)	4,42E-01
GRS QT interval	0,000 (-0,020 - 0,021)	9,79E-01
GRS resting HR	0,005 (-0,015 - 0,025)	6,41E-01
**GRS SBP**	**0,079 (0,059 - 0,100)**	**1,52E-14**
GRS smoking	-0,003 (-0,023 - 0,017)	7,73E-01
GRS TMR during exercise	-0,002 (-0,022 - 0,018)	8,33E-01
GRS TMR during recovery	0,003 (-0,017 - 0,023)	7,58E-01
**GRS resting Tpe**	**-0,022 (-0,042 - -0,002)**	**3,18E-02**
**GRS Triglycerides**	**0,103 (0,082 - 0,123)**	**2,59E-23**

* AF, atrial fibrillation; Beta, effect estimate per standard deviation of the trait for continuous traits; BMI, body mass index; CAD, coronary artery disease; CRP, c-reactive protein; DBP, diastolic blood pressure; GRS, genetic risk score; HDL, high density lipoprotein cholesterol; HF, heart failure; L95, lower limit of the 95% confidence interval of the effect estimate per standard deviation of the trait for continuous traits; LDL, low density lipoprotein cholesterol; MACE, major adverse cardiovascular events; PC, principal component; PP, pulse pressure; SBP, systolic blood pressure; SCD, sudden cardiac death; TMR, T-wave morphology restitution index; Tpe, T-peak-to-T- end interval; U95, upper limit of the 95% confidence interval of the effect estimate per standard deviation of the trait for continuous traits. Significant differences are indicated in bold.

**Table 4:** Risk factors in the scores for MACE

Trait	QRISK3 + CAD GRS		QRISK3 + CAD GRS + CV GRSs		CAD GRS		CAD GRS + CV GRSs	
	Beta (L95 - U95)	P	Beta (L95 - U95)	P	Beta (L95 - U95)	P	Beta (L95 - U95)	P
QRISK3	0,683 (0,668 - 0,697)	1,00E-260	0,681 (0,666 - 0,697)	1,00E-260				
Genetic array [bileve]	0,209 (0,147 - 0,271)	4,13E-11	0,207 (0,145 - 0,269)	6,40E-11	0,315 (0,256 - 0,374)	1,25E-25	0,308 (0,249 - 0,367)	1,78E-24
PC6					-0,035 (-0,057 - -0,013)	1,75E-03	-0,038 (-0,059 - -0,016)	7,83E-04
PC9	0,048 (0,026 - 0,070)	1,99E-05	0,049 (0,027 - 0,071)	1,12E-05	0,045 (0,024 - 0,067)	2,81E-05	0,048 (0,027 - 0,070)	9,13E-06
GRS CAD	0,267 (0,245 - 0,288)	4,27E-134	0,261 (0,240 - 0,283)	6,54E-126	0,284 (0,264 - 0,304)	2,05E-166	0,252 (0,231 - 0,273)	8,30E-123
GRS AF			0,035 (0,013 - 0,056)	1,32E-03			0,045 (0,025 - 0,065)	1,48E-05
GRS BMI			0,023 (0,001 - 0,045)	3,94E-02			0,061 (0,040 - 0,081)	1,06E-08
GRS CRP							0,032 (0,011 - 0,054)	2,73E-03
GRS DBP			0,029 (0,007 - 0,050)	8,35E-03			0,058 (0,038 - 0,079)	2,66E-08
GRS HR response to exercise			0,031 (0,009 - 0,052)	4,67E-03			0,026 (0,006 - 0,047)	1,09E-02
GRS type 2 diabetes			-0,032 (-0,054 - -0,010)	3,97E-03				
GRS HDL			0,043 (0,021 - 0,065)	1,33E-04			-0,040 (-0,063 - -0,017)	5,86E-04
GRS HF			0,072 (0,050 - 0,094)	2,00E-10			0,096 (0,074 - 0,118)	5,55E-18
GRS imaging traits			0,035 (0,014 - 0,057)	1,05E-03			0,029 (0,009 - 0,050)	4,75E-03
GRS LDL							0,044 (0,022 - 0,066)	8,02E-05
GRS PP			0,026 (0,005 - 0,047)	1,70E-02			0,057 (0,037 - 0,078)	4,09E-08
GRS resting Tpe			-0,028 (-0,049 - -0,007)	8,50E-03			-0,028 (-0,048 - -0,007)	7,58E-03
GRS Triglycerides							0,040 (0,016 - 0,063)	8,14E-04

* AF, atrial fibrillation; Beta, effect estimate per standard deviation of the trait for continuous traits; BMI, body mass index; CAD, coronary artery disease; CRP, c-reactive protein; DBP, diastolic blood pressure; GRS, genetic risk score; HDL, high density lipoprotein cholesterol; HF, heart failure; L95, lower limit of the 95% confidence interval of the effect estimate per standard deviation of the trait for continuous traits; LDL, low density lipoprotein cholesterol; MACE, major adverse cardiovascular events; PC, principal component; PP, pulse pressure; SBP, systolic blood pressure; SCD, sudden cardiac death; TMR, T-wave morphology restitution index; Tpe, T-peak-to-T-end interval; U95, upper limit of the 95% confidence interval of the effect estimate per standard deviation of the trait for continuous traits.
