# Supplementary material for: Prediction of Coronary Artery Disease and Major Adverse Cardiovascular Events using Clinical and Genetic Risk Scores for Cardiovascular Risk Factors
Source: Am J Kidney Dis. Author manuscript; Available in PMC 2022 Oct 26. (PMC9584057; doi:10.1053/j.ajkd.2022.01.424)
Supplement: 003441 - Supplemental Material [file EMS146373-supplement-003441___Supplemental_Material.pdf]

SUPPLEMENTAL MATERIAL

Supplemental Methods

Study population

UK Biobank is a prospective study of 502,505 individuals, comprising relatively even numbers of men and women aged 40 to 69 years old at recruitment (2006 - 2008). Individuals were excluded if they were admitted to hospital due to any of the International Classification of Diseases, Tenth Revision (ICD-10) codes in Supplemental Table 1 prior recruitment. Subjects that withdrew from the study, or were of non-European ancestry were also excluded, leading to N = 379,574 individuals included in the analyses (Figure 1).

The primary endpoint of this study was coronary artery disease (CAD) related events, defined as CAD mortality or admission to hospital with a CAD diagnosis (ICD10 codes I21-I23, Supplemental Table 2). The secondary endpoint was major adverse cardiovascular events (MACE). The cause of death was defined according to the *ICD*-10 codes. Date of death was obtained from death certificates held by the National Health Service (NHS) Information Centre and the NHS Central Register Scotland for participants from England and Wales and participants from Scotland, respectively. Diagnoses were captured using the "Spell and Episode" category from the Hospital Episode Statistics records, including both main and secondary diagnoses, coded according to *ICD-10*, made during the hospital inpatient stay. The main diagnoses are those taken to be the main reason for hospital admission, while secondary diagnoses are more often contributory or underlying conditions. Individuals who died from causes not included in the primary or secondary endpoints were censored at the time of death. Follow-up was from the study inclusion date until May 5th, 2021 (median of 11.3 years).

Risk factors

The risk factors used in this study are listed in Supplemental Table 3. First, we derived the QRISK3 score[5] for each participant using the "QRISK" library in R. The model includes body mass index (BMI), family history of heart disease, area deprivation score (Townsend),

smoking intensity, and a number of prevalent conditions including chronic kidney disease stages 3 through 5, atrial fibrillation (AF), migraine, rheumatoid arthritis, systemic lupus erythematosus, mental illness, erectile dysfunction, and antihypertensive medication use. We followed the same definition of each variable described previously[14]. For individuals who reported use of anti-hypertensive medications through questionnaires (Fields 6177 and 6153), their systolic blood pressure (SBP) and diastolic blood pressure (DBP) values were adjusted by adding 15 mmHg and 10 mmHg, respectively, to the mean recording readings[40, 41]. Similarly, for individuals who reported use of lipid-lowering medication through the same questionnaires, their cholesterol and high-density lipoprotein cholesterol were adjusted by dividing by 0.73 and 1.033, respectively, their mean recording readings[14]. We standardized the QRISK3 score to have a mean of 0 and a standard deviation of 1.

In total, 25 genetic risk scores (GRSs) were derived, a GRS for CAD[10] and 24 GRSs for cardiovascular risk factors. These included GRSs for BMI[18], alcohol consumption[16], c-reactive protein[19], SBP, DBP and pulse pressure[20], type 2 diabetes[10], lipids[19], imaging markers[33], AF[10] and heart failure[19]. We also included GRSs for electrocardiogram markers of autonomic tone, including heart rate at rest and heart rate changes during exercise and recovery[22], of atrioventricular conduction (PR interval[23]), ventricular activation (QRS duration), ventricular repolarization risk factors at rest, including the QT interval[25-29] and the T-peak-to-T-end interval[31]. Finally, we included GRSs for ventricular repolarization restitution markers, including QT dynamics during exercise[30] and T-wave morphology restitution during exercise and recovery[32]. A detailed list of traits for which we derived a GRS is described in Supplemental Table 4. PRSice v2[42] was used in this work to derive the GRSs. For this, a list of genetic variants independently associated with each trait was obtained either directly from the corresponding publication (indicated in Supplemental Table 4) or downloaded from the National Human Genome Research Institute-European Bioinformatics Institute GWAS Catalog (https://www.ebi.ac.uk/gwas/), together with the effect sizes (obtained from a replication cohort when possible). PRSice[42] derived GRSs by aggregating all genetic variants

separately for each trait using the effect sizes as independent, unbiased weights. Then, as for QRISK3, the GRS for each trait was standardized to have a mean of 0 and a standard deviation of 1.

As a first step, for each endpoint, CAD and MACE, we split the study population into training (N = 189,787, 50%) and test (N = 189,787, 50%) sets, keeping a balanced case-control ratio and similar distribution of risk factors in both sets (Figure 1). Then, in each training set, we evaluated differences in the risk markers across cases (individuals with the endpoint) and controls (the rest) using Mann-Whitney test.

Building a score combining QRISK3, CAD GRS and GRSs for CV risk factors

In the training set, we first fitted three different models to predict CAD and MACE, respectively: (1) QRISK3, (2) QRISK3 and the CAD GRS, and (3) QRISK3, the CAD GRS and GRSs for the 24 cardiovascular risk factors. Models 2 and 3 were also adjusted for the genetic array and the first ten principal components[9]. For each model, we performed Univariable logistic regression analyses to determine the relationship between each risk factor and CAD or MACE[43]. Then, we took forward into multivariable logistic regression models those risk factors significantly associated with the endpoints ($P < 5 \times 10^{-2}$) using backward stepwise elimination to remove markers with a non-significant association with the endpoint.

Risk scores were calculated as the weighted sum of significant risk markers in the final multivariable models from the training set, weighted by the corresponding beta coefficients[43-45]. Performance was evaluated by measuring the concordance index (C index) for individuals in the test set. The C index of two different scores was compared with a Student t test for paired samples using the library "pROC" in R.

Next, for each score and endpoint, we identified two risk groups based on their training-specific optimal cutoff, calculated as the value of the score that jointly maximized both sensitivity and specificity values (using the "cutpointr" package in R). Thus, both risk groups were defined as: low-risk (test score values < optimal cutoff) and high-risk (test score values > optimal cutoff).

Odds ratios (ORs) were calculated using the low-risk group as a reference. The net reclassification improvement (NRI)[46] was used to quantify the added predictive value of each score beyond that from the corresponding preceding one for both CAD and MACE. We predicted risk after median follow-up (4,155 days) and bootstrapping (100 iterations) was performed to derive confidence intervals. The risk categories used for the NRI analysis were 5%, 5 to 10% and ≥10%[47]. A version of NRI appropriate for survival analyses was computed using the Kaplan–Meier method[46]. NRI was computed using the package “nricens” in R.

Building a score combining CAD GRS and GRSs for CV risk factors

We also repeated the analyses by fitting two additional models for CAD and MACE when not taking QRISK3 into account: (4) the CAD GRS, and (5) the CAD GRS and GRSs for the 24 cardiovascular risk factors. Both models were adjusted for the genetic array and the first ten principal components[9]. For both models we followed the same steps described in the section above.

Supplemental Tables

Supplemental Table I: Codes used to define the MACE group

**Myocardial Infarction**		
	**ICD10 codes**	**Definition**
**Myocardial Infarction**	I21	Acute myocardial infarction
	I21.0	Acute transmural myocardial infarction of anterior wall
	I21.1	Acute transmural myocardial infarction of inferior wall
	I21.2	Acute transmural myocardial infarction of other sites
	I21.3	Acute transmural myocardial infarction of unspecified site
	I21.4	Acute subendocardial myocardial infarction
	I21.9	Acute myocardial infarction, unspecified
	I22	Subsequent myocardial infarction
	I22.0	Subsequent myocardial infarction of anterior wall
	I22.1	Subsequent myocardial infarction of inferior wall
	I22.8	Subsequent myocardial infarction of other sites
	I22.9	Subsequent myocardial infarction of unspecified site
	I23	Certain current complications following acute myocardial infarction
	I23.0	Haemopericardium as current complication following acute myocardial infarction
	I23.1	Atrial septal defect as current complication following acute myocardial infarction
	I23.2	Ventricular septal defect as current complication following acute myocardial infarction
	I23.3	Rupture of cardiac wall without haemopericardium as current complication following acute myocardial infarction
	I23.4	Rupture of chordae tendineae as current complication following acute myocardial infarction

I23.5	Rupture of papillary muscle as current complication following acute myocardial infarction
I23.6	Thrombosis of atrium , auricular appendage and ventricle as current complications following acute myocardial infarction
I23.8	Other current complications following acute myocardial infarction
**ICD9 codes**	**Definition**
4109	Acute myocardial infarction
**Operation (self-reported)**	**Definition**
1070	Coronary angioplasty (ptca) + stent
1095	Coronary artery bypass grafts (cabg)
1523	Triple Heart bypass
**OPCS4**	**Definition**
K40	Saphenous vein graft replacement of coronary artery
K40.1	Saphenous vein graft replacement of one coronary artery
K40.2	Saphenous vein graft replacement of two coronary arteries
K40.3	Saphenous vein graft replacement of three coronary arteries
K40.4	Saphenous vein graft replacement of four or more coronary arteries
K40.9	Unspecified saphenous vein graft replacement of coronary artery
K41	Other autograft replacement of coronary artery
K41.1	Autograft replacement of one coronary artery NEC
K41.2	Autograft replacement of two coronary arteries NEC
K41.3	Autograft replacement of three coronary arteries NEC
K41.4	Autograft replacement of four or more coronary arteries NEC
K42	Allograft replacement of coronary artery
K42.4	Allograft replacement of four or more coronary arteries

K44	Other replacement of coronary artery
K44.1	Replacement of coronary arteries using multiple methods
K44.2	Revision of replacement of coronary artery
K44.9	Unspecified other replacement of coronary artery
K45	Connection of thoracic artery to coronary artery
K45.1	Double anastomosis of mammary arteries to coronary arteries
K45.2	Double anastomosis of thoracic arteries to coronary arteries NEC
K45.3	Anastomosis of mammary artery to left anterior descending coronary artery
K45.4	Anastomosis of mammary artery to coronary artery NEC
K45.5	Anastomosis of thoracic artery to coronary artery NEC
K45.6	Revision of connection of thoracic artery to coronary artery
K45.8	Other specified connection of thoracic artery to coronary artery
K45.9	Unspecified connection of thoracic artery to coronary artery
K49	Transluminal balloon angioplasty of coronary artery
K49.1	Percutaneous transluminal balloon angioplasty of one coronary artery
K49.2	Percutaneous transluminal balloon angioplasty of multiple coronary arteries
K49.3	Percutaneous transluminal balloon angioplasty of bypass graft of coronary artery
K49.4	Percutaneous transluminal cutting balloon angioplasty of coronary artery
K49.8	Other specified transluminal balloon angioplasty of coronary artery
K49.9	Unspecified transluminal balloon angioplasty of coronary artery
K50	Other therapeutic transluminal operations on coronary artery
K50.1	Percutaenous transluminal laser coronary angioplasty

	K50.2	Percutaneous transluminal coronary thrombolysis using streptokinase
	K50.3	Percutaneous transluminal injection of therapeutic substance into coronary artery NEC
	K50.4	Percutaenous transluminal atherectomy of coronary artery
	K50.8	Other specified other therapeutic transluminal operations on coronary artery
	K50.9	Unspecified other therapeutic transluminal operations on coronary artery
	K75	Percutaneous transluminal balloon angioplasty and insertion of stent into coronary artery
	K75.1	Percutaneous transluminal balloon angioplasty and insertion of 1-2 drug-eluting stents into coronary artery
	K75.2	Percutaneous transluminal balloon angioplasty and insertion of 3 or more drug-eluting stents into coronary artery
	K75.3	Percutaneous transluminal balloon angioplasty and insertion of 1-2 stents into coronary artery
	K75.4	Percutaneous transluminal balloon angioplasty and insertion of 3 or more stents into coronary artery NEC
	K75.8	Other specified percutaenous transluminal balloon angioplasty and insertion of stent into coronary artery
	K75.9	Unspecified percutaenous transluminal balloon angioplasty and insetion of stent into coronary artery
	**Heart Failure**	
**Heart Failure**	**ICD10 codes**	**Definition**
	I13.0	Hypertensive heart and renal disease with both (congestive) heart failure

	I13.2	Hypertensive heart and renal disease with both (congestive) heart failure and renal failure
	I50	Heart failure
	I50.0	Congestive heart failure
	I50.1	Left ventricular failure
	I50.9	Heart failure, unspecified
	**ICD9 codes**	**Definition**
	4280	Congestive heart failure
	4281	Left heart failure
	4289	Heart failure, unspecified
	**OPCS4**	**Definition**
	K59.6	Implantation of cardioverter defibrillator using three electrode leads
	K61.7	Implantation of biventricular cardiac pacemaker system
	K60.7	Implantation of intravenous biventricular cardiac pacemaker system
**Ventricular Arrhythmia**		**Ventricular Arrhythmia**
	**ICD10 codes**	**Definition**
	I47.2	Ventricular tachycardia
	I49.0	Ventricular fibrillation and flutter
	I46.0	Cardiac arrest with successful resuscitation
	I46.1	Sudden cardiac death, so described
	I46.9	Cardiac arrest, unspecified
	I47.0	Re-entry ventricular arrhythmia
	K57.6	
	K64.1	Second degree haemorrhoids
	X50.3	Overexertion from repetitive movements
	X50.4	
		**ICD Implant**

	OPCS4	Definition
ICD Implant	K59	Cardioverter defibrillator introduced through vein
	K59.1	Implantation of cardioverter defibrillator using one electrode lead
	K59.2	Implantation of cardioverter defibrillator using two electrode leads
	K59.3	Resitting of lead of cardioverter defibrillator
	K59.4	Renewal of cardioverter defibrillator
	K59.6	Implantation of cardioverter defibrillator using three electrode leads
	K59.8	Other specified cardioverter defibrillator introduced through the vein
	K59.9	Unspecified cardioverter defibrillator introduced through the vein
	K72	Other cardioverter defibrillator
	K72.1	Implantation of subcutaneous cardioverter defibrillator
	K72.3	Renewal of subcutaneous cardioverter defibrillator

Supplemental Table II: Codes used to define the CAD group

ICD10 codes	Definition
I21	Acute myocardial infarction
I21.0	Acute transmural myocardial infarction of anterior wall
I21.1	Acute transmural myocardial infarction of inferior wall
I21.2	Acute transmural myocardial infarction of other sites
I21.3	Acute transmural myocardial infarction of unspecified site
I21.4	Acute subendocardial myocardial infarction
I21.9	Acute myocardial infarction, unspecified
I22	Subsequent myocardial infarction
I22.0	Subsequent myocardial infarction of anterior wall
I22.1	Subsequent myocardial infarction of inferior wall
I22.8	Subsequent myocardial infarction of other sites
I22.9	Subsequent myocardial infarction of unspecified site
I23	Certain current complications following acute myocardial infarction
I23.0	Hemopericardium as current complication following acute myocardial infarction
I23.1	Atrial septal defect as current complication following acute myocardial infarction
I23.2	Ventricular septal defect as current complication following acute myocardial infarction
I23.3	Rupture of cardiac wall without hemopericardium as current complication following acute myocardial infarction
I23.4	Rupture of chordae tendineae as current complication following acute myocardial infarction
I23.5	Rupture of papillary muscle as current complication following acute myocardial infarction

I23.6	Thrombosis of atrium , auricular appendage and ventricle as current complications following acute myocardial infarction
I23.8	Other current complications following acute myocardial infarction
**ICD9 codes**	**Definition**
4109	Acute myocardial infarction
**Operation (self-reported)**	**Definition**
1070	Coronary angioplasty (ptca) + stent
1095	Coronary artery bypass grafts (cabg)
1523	Triple Heart bypass
**OPCS4**	**Definition**
K40	Saphenous vein graft replacement of coronary artery
K40.1	Saphenous vein graft replacement of one coronary artery
K40.2	Saphenous vein graft replacement of two coronary arteries
K40.3	Saphenous vein graft replacement of three coronary arteries
K40.4	Saphenous vein graft replacement of four or more coronary arteries
K40.9	Unspecified saphenous vein graft replacement of coronary artery
K41	Other autograft replacement of coronary artery
K41.1	Autograft replacement of one coronary artery NEC
K41.2	Autograft replacement of two coronary arteries NEC
K41.3	Autograft replacement of three coronary arteries NEC
K41.4	Autograft replacement of four or more coronary arteries NEC
K42	Allograft replacement of coronary artery
K42.4	Allograft replacement of four or more coronary arteries
K44	Other replacement of coronary artery
K44.1	Replacement of coronary arteries using multiple methods
K44.2	Revision of replacement of coronary artery

K44.9	Unspecified other replacement of coronary artery
K45	Connection of thoracic artery to coronary artery
K45.1	Double anastomosis of mammary arteries to coronary arteries
K45.2	Double anastomosis of thoracic arteries to coronary arteries NEC
K45.3	Anastomosis of mammary artery to left anterior descending coronary artery
K45.4	Anastomosis of mammary artery to coronary artery NEC
K45.5	Anastomosis of thoracic artery to coronary artery NEC
K45.6	Revision of connection of thoracic artery to coronary artery
K45.8	Other specified connection of thoracic artery to coronary artery
K45.9	Unspecified connection of thoracic artery to coronary artery
K49	Transluminal balloon angioplasty of coronary artery
K49.1	Percutaneous transluminal balloon angioplasty of one coronary artery
K49.2	Percutaneous transluminal balloon angioplasty of multiple coronary arteries
K49.3	Percutaneous transluminal balloon angioplasty of bypass graft of coronary artery
K49.4	Percutaneous transluminal cutting balloon angioplasty of coronary artery
K49.8	Other specified transluminal balloon angioplasty of coronary artery
K49.9	Unspecified transluminal balloon angioplasty of coronary artery
K50	Other therapeutic transluminal operations on coronary artery
K50.1	Percutaneous transluminal laser coronary angioplasty
K50.2	Percutaneous transluminal coronary thrombolysis using streptokinase
K50.3	Percutaneous transluminal injection of therapeutic substance into coronary artery NEC
K50.4	Percutaneous transluminal atherectomy of coronary artery

K50.8	Other specified other therapeutic transluminal operations on coronary artery
K50.9	Unspecified other therapeutic transluminal operations on coronary artery
K75	Percutaneous transluminal balloon angioplasty and insertion of stent into coronary artery
K75.1	Percutaneous transluminal balloon angioplasty and insertion of 1-2 drug-eluting stents into coronary artery
K75.2	Percutaneous transluminal balloon angioplasty and insertion of 3 or more drug-eluting stents into coronary artery
K75.3	Percutaneous transluminal balloon angioplasty and insertion of 1-2 stents into coronary artery
K75.4	Percutaneous transluminal balloon angioplasty and insertion of 3 or more stents into coronary artery NEC
K75.8	Other specified percutaneous transluminal balloon angioplasty and insertion of stent into coronary artery
K75.9	Unspecified percutaneous transluminal balloon angioplasty and insertion of stent into coronary artery

Supplemental Table III: Characteristics of individuals according to their endpoint.

	CAD				MACE			
**Variable**	**All N = 189,787**	**Cases N = 6,186**	**Controls N = 183,601**	***P***	**All N = 189,787**	**Cases N = 9,900**	**Controls N = 179,887**	***P***
**QRISK3**	-0.280 (1.137)	0.658 (1.652)	-0.304 (1.104)	**<2.2E-16**	-0.280 (1.137)	0.722 (1.762)	-0.319 (1.080)	**<2.2E-16**
**GRS for CAD [SD]**	-0.007 (1.335)	0.420 (1.353)	-0.021 (1.331)	**<2.2E-16**	-0.007 (1.335)	0.270 (1.365)	-0.021 (1.331)	**<2.2E-16**
GRS for AF [SD]	-0.042 (1.330)	-0.030 (1.333)	-0.043 (1.330)	8.70E-02	-0.042 (1.330)	0.009 (1.330)	-0.045 (1.329)	**1.17E-15**
GRS for alcohol consumption [SD]	-0.001 (1.349)	-0.023 (1.347)	0.000 (1.350)	**2.10E-02**	-0.001 (1.349)	-0.002 (1.338)	-0.001 (1.350)	9.09E-01
GRS for BMI [SD]	-0.001 (1.352)	0.052 (1.326)	-0.003 (1.353)	**1.31E-08**	-0.001 (1.352)	0.077 (1.336)	-0.005 (1.353)	**<2.2E-16**
GRS for c-reactive protein [SD]	0.013 (1.353)	0.049 (1.362)	0.012 (1.353)	**1.43E-04**	0.013 (1.353)	0.069 (1.355)	0.010 (1.353)	**3.44E-15**
GRS for DBP [SD]	-0.004 (1.346)	0.089 (1.320)	-0.007 (1.347)	**<2.2E-16**	-0.004 (1.346)	0.066 (1.334)	-0.007 (1.347)	**<2.2E-16**
GRS for HR dynamics during exercise [SD]	-0.009 (1.348)	-0.007 (1.336)	-0.009 (1.348)	8.90E-01	-0.009 (1.348)	0.006 (1.332)	-0.009 (1.349)	7.31E-02
GRS for HR dynamics during recovery [SD]	0.023 (1.365)	0.034 (1.347)	0.023 (1.365)	6.10E-01	0.023 (1.365)	0.034 (1.341)	0.022 (1.366)	4.91E-01
GRS for type 2 diabetes [SD]	-0.007 (1.344)	0.053 (1.360)	-0.009 (1.343)	**2.75E-13**	-0.007 (1.344)	0.045 (1.350)	-0.010 (1.343)	**2.38E-13**
GRS for QT dynamics during exercise [SD]	-0.006 (1.323)	0.010 (1.317)	-0.006 (1.324)	3.08E-01	-0.006 (1.323)	0.014 (1.331)	-0.007 (1.323)	**2.06E-02**
GRS for HDL [SD]	0.003 (1.351)	-0.142 (1.365)	0.007 (1.350)	**<2.2E-16**	0.003 (1.351)	-0.107 (1.352)	0.008 (1.350)	**<2.2E-16**
GRS for HF [SD]	-0.000 (1.350)	0.149 (1.363)	-0.005 (1.350)	**<2.2E-16**	-0.000 (1.350)	0.141 (1.384)	-0.008 (1.348)	**<2.2E-16**
GRS for imaging traits [SD]	0.026 (1.335)	0.045 (1.308)	0.026 (1.336)	7.27E-02	0.026 (1.335)	0.063 (1.322)	0.024 (1.336)	**7.38E-07**
GRS for LDL [SD]	0.049 (1.325)	0.225 (1.267)	0.043 (1.326)	**<2.2E-16**	0.049 (1.325)	0.153 (1.295)	0.043 (1.326)	**<2.2E-16**
GRS for PP [SD]	0.001 (1.349)	0.102 (1.354)	-0.003 (1.349)	**<2.2E-16**	0.001 (1.349)	0.076 (1.357)	-0.004 (1.349)	**<2.2E-16**

GRS for PR interval [SD]	0.004 (1.343)	-0.003 (1.338)	0.005 (1.343)	7.34E-01	0.004 (1.343)	-0.016 (1.350)	0.006 (1.342)	**5.92E-03**
GRS for QRS duration [SD]	-0.008 (1.349)	-0.009 (1.339)	-0.008 (1.349)	7.73E-01	-0.008 (1.349)	-0.017 (1.330)	-0.007 (1.350)	**4.15E-02**
GRS for QT interval [SD]	-0.004 (1.338)	-0.006 (1.359)	-0.004 (1.337)	8.49E-01	-0.004 (1.338)	-0.010 (1.363)	-0.003 (1.337)	9.34E-01
GRS for resting HR [SD]	-0.005 (1.351)	-0.000 (1.333)	-0.006 (1.352)	5.68E-01	-0.005 (1.351)	-0.002 (1.335)	-0.006 (1.352)	9.60E-01
GRS for SBP [SD]	-0.001 (1.349)	0.093 (1.342)	-0.003 (1.350)	**<2.2E-16**	-0.001 (1.349)	0.078 (1.345)	-0.005 (1.349)	**<2.2E-16**
GRS for smoking [SD]	-0.002 (1.357)	-0.008 (1.328)	-0.002 (1.357)	8.12E-01	-0.002 (1.357)	-0.002 (1.336)	-0.002 (1.360)	2.62E-01
GRS for TMR during exercise [SD]	-0.009 (1.315)	-0.001 (1.289)	-0.009 (1.316)	7.48E-01	-0.009 (1.315)	0.000 (1.312)	-0.010 (1.315)	8.86E-01
GRS for TMR during recovery [SD]	0.099 (1.060)	0.099 (1.081)	0.099 (1.060)	9.30E-01	0.099 (1.060)	0.102 (1.071)	0.099 (1.059)	1.73E-01
GRS for resting Tpe interval [SD]	0.012 (1.347)	-0.000 (1.342)	0.012 (1.348)	1.30E-01	0.012 (1.347)	-0.011 (1.358)	0.013 (1.347)	**7.34E-03**
GRS for triglycerides [SD]	-0.011 (1.345)	0.132 (1.338)	-0.016 (1.345)	**<2.2E-16**	-0.011 (1.345)	0.088 (1.353)	-0.017 (1.345)	**<2.2E-16**

Data are represented as median (interquartile range)

BMI, body mass index; bpm, beats per minute; CAD, coronary artery disease; DBP, diastolic blood pressure; GRS, genetic risk score; HDL, high density lipoprotein cholesterol; HF, heart failure; HR, heart rate; LDL, low density lipoprotein cholesterol; MACE, major adverse cardiovascular events; PP, pulse pressure; SBP, systolic blood pressure; SD, standard deviation; TMR, T-wave morphology restitution index; Tpe, T-peak-to-T-end interval;

Supplemental Table IV: Details of the original studies reporting the variants used to derive the GRSs.

Trait	N variants	PMID	First author	Year
Type 2 diabetes	6,917,436	30104762	Khera	2018
Smoker	28	30617275	Erzurumluoglu	2019
Alcohol drinker	1,099,480	32555147	Barr	2020
BMI	2,100,302	31002795	Khera	2019
CRP	17,378	33462484	Sinnott-Armstrong	2021
SBP	570	30224653	Evangelou	2018
DBP	576	30224653	Evangelou	2018
PP	492	30224653	Evangelou	2018
HDL	25,069	33462484	Sinnott-Armstrong	2021
LDL	16,184	33462484	Sinnott-Armstrong	2021
triglycerides	16,003	33462484	Sinnott-Armstrong	2021
Resting HR	458	33679875	Mensah-Kane	2020
HR Response to exercise	14	29769521	Ramírez	2018
HR Response to recovery	16	29769521	Ramírez	2018
PR	583	32439900	Ntalla	2020
QRS	52	27659466	van der Harst	2016
QTc	68	19305408, 20031603, 30679814, 24952745, 34274964	Newton Cheh, Marroni, van Setten, Arking, van Duijvenboden	2009, 2009, 2019, 2014, 2021
QT dynamics during exercise	19	32527199	van Duijvenboden	2020
Tpe interval	28	32386560	Ramírez	2020
TMRex	8	31607149	Ramírez	2019
TMRrec	8	31607149	Ramírez	2019
Imaging	28	32382064	Pirruccello	2020
CAD	6,630,150	30104762	Khera	2018
AF	6,730,541	30104762	Khera	2018
HF	183,287	33462484	Sinnott-Armstrong	2021

AF, atrial fibrillation; BMI, body mass index; CAD, coronary artery disease; CRP, c-reactive protein; DBP, diastolic blood pressure; HDL, high-density lipoprotein cholesterol; HF, heart failure; HR, heart rate; LDL, low density lipoprotein cholesterol; PP, pulse pressure; QTc,

corrected QT interval; SBP, systolic blood pressure; TMR, T-wave morphology restitution index; Tpe, T-peak-to-T-end

Supplemental Table V: Net reclassification improvement for CAD for the score including QRISK3 and the CAD GRS versus QRISK3 alone with estimates of the expected number of reclassifications per risk category for cases and controls

Reclassification Table for all subjects

	QRISK3 + CAD GRS					
**QRISK3**	**1 to <5%**	**5 to <10%**	**≥ 10 %**	**Reclassified up, n(%)**	**Reclassified down, n(%)**	**NRI (95% CI)**
**<5%**	162,433	8,001	289	10,092 (5.3)	6,039 (3.2)	0.077 (0.075 - 0.079)
**5 to <10%**	4,840	7,388	1,802			
**≥ 10%**	77	1,122	3,835			

Reclassification Table for cases

	QRISK3 + CAD GRS					
**QRISK3**	**1 to <5%**	**5 to <10%**	**≥ 10 %**	**Reclassified up, n(%)**	**Reclassified down, n(%)**	**NRI (95% CI)**
**<5%**	3,095	644	29	931 (15.1)	390 (6.3)	0.096 (0.094 - 0.099)
**5 to <10%**	285	638	258			
**≥ 10%**	4	101	600			

Reclassification Table for controls

	QRISK3 + CAD GRS			Reclassified up, n(%)	Reclassified down, n(%)	NRI (95% CI)
**QRISK3**	**1 to <5%**	**5 to <10%**	**≥ 10 %**			
**<5%**	142,926	6,281	218	7,727 (4.2)	4,525 (2.5)	-0.019 (-0.019 - -0.019)
**5 to <10%**	3,709	5,413	1,228			
**≥ 10%**	53	763	2,417			

Abbreviations: NRI: Net reclassification index, CI: confidence interval. The score including QRISK3 and the CAD GRS is also adjusted for the genetic array and first 10 principal components.

Supplemental Table VI: Net reclassification improvement for CAD for the score including QRISK3 and the CAD GRS versus QRISK3 alone with estimates of the expected number of reclassifications per risk category for cases and controls

Reclassification Table for all subjects

	QRISK3 + CAD GRS			Reclassified up, n(%)	Reclassified down, n(%)	NRI (95% CI)
**QRISK3**	**1 to <5%**	**5 to <10%**	**≥ 10%**			
**<5%**	127,994	10,935	39	14,238 (7.5)	10,798 (5.7)	0.039 (0.038 - 0.041)
**5 to <10%**	8,428	24,917	3,264			
**≥ 10%**	9	2,361	11,840			

Reclassification Table for cases

	QRISK3 + CAD GRS			Reclassified up, n(%)	Reclassified down, n(%)	NRI (95% CI)
**QRISK3**	**1 to <5%**	**5 to <10%**	**≥ 10%**			
**<5%**	2,563	754	6	1,246 (12.6)	735 (7.4)	0.055 (0.054 - 0.057)
**5 to <10%**	464	2,162	486			
**≥ 10%**	1	270	2,351			

Reclassification Table for controls

QRISK3	QRISK3 + CAD GRS			Reclassified up, n(%)	Reclassified down, n(%)	NRI (95% CI)
	**1 to <5%**	**5 to <10%**	**≥ 10%**			
**<5%**	66,957	5,211	16	6,580 (3.7)	4,913 (2.7)	-0.016 (-0.016 - -0.016)
**5 to <10%**	3,924	10,962	1,353			
**≥ 10%**	2	987	4,368			

Abbreviations: NRI: Net reclassification index, CI: confidence interval. The score including QRISK3 and the CAD GRS is also adjusted for the genetic array and first 10 principal components.

Supplemental Table VII: Net reclassification improvement for CAD for the score combining the CAD GRS and GRSs for multiple CV risk factors versus the CAD GRS alone with estimates of the expected number of reclassifications per risk category for cases and controls

Reclassification Table for all subjects

	CAD GRS + GRSs for multiple CV risk factors			Reclassified up, n(%)	Reclassified down, n(%)	NRI (95% CI)
**CAD GRS**	**1 to <5%**	**5 to <10%**	**≥ 10%**			
**<5%**	166,500	7,690	4	8,099 (4.3)	4,447 (2.3)	0.033 (0.031 - 0.035)
**5 to <10%**	4,334	10,541	405			
**≥ 10%**	0	113	200			

Reclassification Table for cases

	CAD GRS + GRSs for multiple CV risk factors			Reclassified up, n(%)	Reclassified down, n(%)	NRI (95% CI)
**CAD GRS**	**1 to <5%**	**5 to <10%**	**≥ 10%**			
**<5%**	4,282	437	0	484 (7.8)	189 (3.1)	0.051 (0.049 - 0.053)
**5 to <10%**	180	678	47			
**≥ 10%**	0	9	21			

Reclassification Table for controls

	CAD GRS + GRSs for multiple CV risk factors			Reclassified up, n(%)	Reclassified down, n(%)	NRI (95% CI)
**CAD GRS**	**1 to <5%**	**5 to <10%**	**≥ 10%**			
**<5%**	143,609	6,439	3	6,754 (3.7)	3777 (2.1)	-0.018 (-0.019 - -0.017)
**5 to <10%**	3,689	8,704	312			
**≥ 10%**	0	88	164			

Abbreviations: NRI: Net reclassification index, CI: confidence interval, CV cardiovascular

Both scores were adjusted for the genetic array and first 10 principal components.

Supplemental Table VIII: Net reclassification improvement for MACE for the score combining the CAD GRS and GRSs for multiple CV risk factors versus the CAD GRS alone with estimates of the expected number of reclassifications per risk category for cases and controls

Reclassification Table for all subjects

	CAD GRS + GRSs for multiple CV risk factors			Reclassified up, n(%)	Reclassified down, n(%)	NRI (95% CI)
**CAD GRS**	**1 to <5%**	**5 to <10%**	**≥ 10%**			
**<5%**	81,360	18,819	18	21,357 (11.3)	23,078 (12.2)	0.038 (0.036 - 0.040)
**5 to <10%**	22,703	63,490	2,520			
**≥ 10%**	0	375	502			

Reclassification Table for cases

	CAD GRS + GRSs for multiple CV risk factors			Reclassified up, n(%)	Reclassified down, n(%)	NRI (95% CI)
**CAD GRS**	**1 to <5%**	**5 to <10%**	**≥ 10%**			
**<5%**	2,795	973	1	1,247 (12.6)	1,023 (10.3)	0.027 (0.025 - 0.028)
**5 to <10%**	987	3,936	273			
**≥ 10%**	0	36	56			

Reclassification Table for controls

	CAD GRS + GRSs for multiple CV risk factors			Reclassified up, n(%)	Reclassified down, n(%)	NRI (95% CI)
**CAD GRS**	**1 to <5%**	**5 to <10%**	**≥ 10%**			
**<5%**	40,585	9,350	8			
**5 to <10%**	11,345	30,865	1,218	10,576 (5.9)	11,523 (6.4)	0.011 (0.010 - 0.012)
**≥ 10%**	0	178	231			

Abbreviations: NRI: Net reclassification index, CI: confidence interval. Both scores were adjusted for the genetic array and first 10 principal components.
